# Signatures of ecological processes in microbial community time series

**DOI:** 10.1186/s40168-018-0496-2

**Published:** 2018-06-28

**Authors:** Karoline Faust, Franziska Bauchinger, Béatrice Laroche, Sophie de Buyl, Leo Lahti, Alex D. Washburne, Didier Gonze, Stefanie Widder

**Affiliations:** 1 KU Leuven Department of Microbiology and Immunology, Rega Institute, Laboratory of Molecular Bacteriology, Leuven, Belgium; 2 Division of Microbial Ecology, Department of Microbiology and Ecosystem Sciences, University of Vienna, Althanstr. 14, 1090 Vienna, Austria; 3 Département de Mathématiques Informatiques Appliquées, INRA, Jouy-en-Josas, France; 4 Applied Physics, Vrije Universiteit Brussel, Pleinlaan 2, 1050 Brussels, Belgium; 5 Interuniversity Institute of Bioinformatics in Brussels, ULB/VUB, Triomflaan, 1050 Brussels, Belgium; 6 VIB Center for the Biology of Disease, Herestraat 49, 3000 Leuven, Belgium; 7 Department of Mathematics and Statistics, University of Turku, 20014 Turku, Finland; 8 Department of Biology, Duke University, Durham, NC USA; 9 Department of Microbiology and Immunology, Montana State University, Bozeman, MT USA; 10 Unité de Chronobiologie Théorique, Faculté des Sciences, Université Libre de Bruxelles, Bvd du Triomphe, 1050 Brussels, Belgium; 11 CeMM-Reseach Center for Molecular Medicine of the Austrian Academy of Sciences, Lazarettgasse 14, 1090 Vienna, Austria; 12 Department of Medicine 1, Research Laboratory of Infection Biology, Medical University of Vienna, Währinger Gürtel 18-20, 1090 Vienna, Austria; 13 Konrad Lorenz Institute for Evolution and Cognition Research, Martinstr. 12, 4300 Klosterneuburg, Austria

**Keywords:** Noise types, Community dynamics, Community models, Time series analysis, Neutrality test, Pink noise, Brown noise

## Abstract

**Background:**

Growth rates, interactions between community members, stochasticity, and immigration are important drivers of microbial community dynamics. In sequencing data analysis, such as network construction and community model parameterization, we make implicit assumptions about the nature of these drivers and thereby restrict model outcome. Despite apparent risk of methodological bias, the validity of the assumptions is rarely tested, as comprehensive procedures are lacking. Here, we propose a classification scheme to determine the processes that gave rise to the observed time series and to enable better model selection.

**Results:**

We implemented a three-step classification scheme in R that first determines whether dependence between successive time steps (temporal structure) is present in the time series and then assesses with a recently developed neutrality test whether interactions between species are required for the dynamics. If the first and second tests confirm the presence of temporal structure and interactions, then parameters for interaction models are estimated. To quantify the importance of temporal structure, we compute the noise-type profile of the community, which ranges from black in case of strong dependency to white in the absence of any dependency. We applied this scheme to simulated time series generated with the Dirichlet-multinomial (DM) distribution, Hubbell’s neutral model, the generalized Lotka-Volterra model and its discrete variant (the Ricker model), and a self-organized instability model, as well as to human stool microbiota time series. The noise-type profiles for all but DM data clearly indicated distinctive structures. The neutrality test correctly classified all but DM and neutral time series as non-neutral. The procedure reliably identified time series for which interaction inference was suitable. Both tests were required, as we demonstrated that all structured time series, including those generated with the neutral model, achieved a moderate to high goodness of fit to the Ricker model.

**Conclusions:**

We present a fast and robust scheme to classify community structure and to assess the prevalence of interactions directly from microbial time series data. The procedure not only serves to determine ecological drivers of microbial dynamics, but also to guide selection of appropriate community models for prediction and follow-up analysis.

**Electronic supplementary material:**

The online version of this article (10.1186/s40168-018-0496-2) contains supplementary material, which is available to authorized users.

## Background

Microbial communities perform essential ecosystem services and carry out important functions in their hosts. For instance, the healthy human gut microbiome protects its host from pathogens, expands the host’s digestive capacities and contributes to human immune system maturation [1]. Thanks to recent advances in sequencing technology, we now have access to data sets that capture the dynamics of entire microbial communities at a high phylogenetic resolution over long periods of time. Such densely sampled long-term time series data have been collected for instance for skin and gut [2, 3], but also for lakes and oceans [4, 5]. These studies have illustrated that proportions of microbial taxa do not always remain constant, but can vary over time, sometimes considerably so, for instance in cases of seasonality [5] and succession [6].

Fluctuations in microbial community composition have been linked to a variety of inter-dependent factors, including ecological interactions between community members, environmental conditions, immigration from adjacent ecosystems, the history of the community, and the evolution of community members [7, 8]. The importance of these factors varies across ecosystems; hence, a better characterization of their contribution will enable selecting suitable community models and improve our understanding of microbial community functions.

A popular strategy to learn more about microbial community structure and dynamics is to fit community models to sequencing data of microbial communities and to analyze the parameterized models. Two frequently selected community models are Hubbell’s neutral model [9, 10] and the generalized Lotka-Volterra (gLV) model [11, 12], where the former assumes that species are ecologically equivalent and community dynamics is governed by local extinction and immigration from a metacommunity, whereas the latter describes the change of species abundances as a function of growth rates and species interactions. The parameters (and therefore the species interactions) of the deterministic gLV model and its stochastic, time discrete version, the Ricker model, have been inferred directly from time series data by several authors (e.g., [13–18]). The continuous form of the Hubbell model developed by Sloan [19] served as a null model against which over- or under-representation of microbial taxa was tested [20, 21]. The self-organized instability (SOI) model [22] proposed by Solé and colleagues combines aspects of the gLV and neutral model, namely interactions with stochastic immigration and extinction. Alternative ways to integrate interactions and immigration have also been suggested [23–26].

**Fig. 1:**
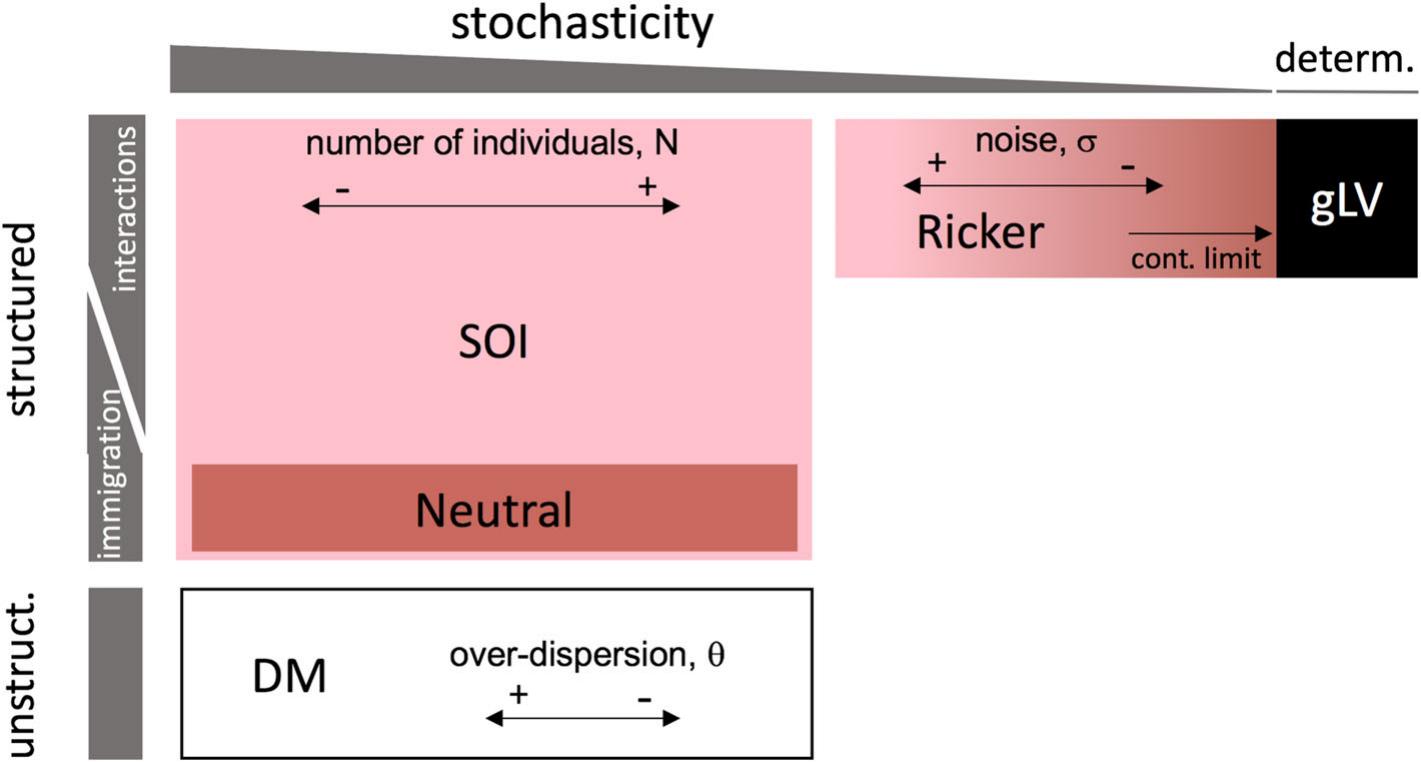
Overview of community models. The position of community models in this overview diagram is determined by two axes, which represent the importance given to structure and to noise, respectively. The first gradient orders models by the level of stochasticity, with neutral models at one extreme and the noise-free Ricker and generalized Lotka-Volterra model at the other. When increasing the strength of the noise or decreasing the number of individuals, deterministic models can move towards the stochastic end of the spectrum. The second axis orders models according to the role of structure, i.e., the strength of the dependency on previous time points. The Dirichlet-multinomial distribution and other probability distributions, which generate counts that do not depend on previous states, are at one end of the spectrum, whereas models with a high dependency on previous states, such as the generalized Lotka-Volterra, are at the other

Although these models emphasize different aspects of community dynamics, they can be seen as realizations of an encompassing community model framework spanning a gradient from stochastic to deterministic and from unstructured to temporally structured in time (Fig. 1). Temporal structure is defined here in the sense of a Markovian structure, which means that the state of the system at a certain time point depends on its state at preceding time points. Structured models, which include all models considered here except the DM, encode rules that describe how the community composition changes from one time step to the next. In contrast, unstructured models, which are stochastic, successively sample independent microbial community composition from a probability distribution, such as the Dirichlet-multinomial distribution [27, 28].

Temporally structured stochastic models, such as the neutral and the SOI model, describe microbial community dynamics at the level of individuals. With increasing number of individuals, their behavior approaches that of deterministic models describing community dynamics at the level of populations, such as the gLV and its discrete variant, the Ricker model [14]. These deterministic models in turn can move towards the stochastic end of the gradient by integrating intrinsic noise or random environmental fluctuations of increasing strength.

In this work, we aim to provide guidelines that help distinguish the different generating processes directly from the time series, thereby complementing and guiding standard model fitting procedures. More precisely, our goal is to determine whether the inference of a microbial network from a time series data set is meaningful.

**Fig. 2:**
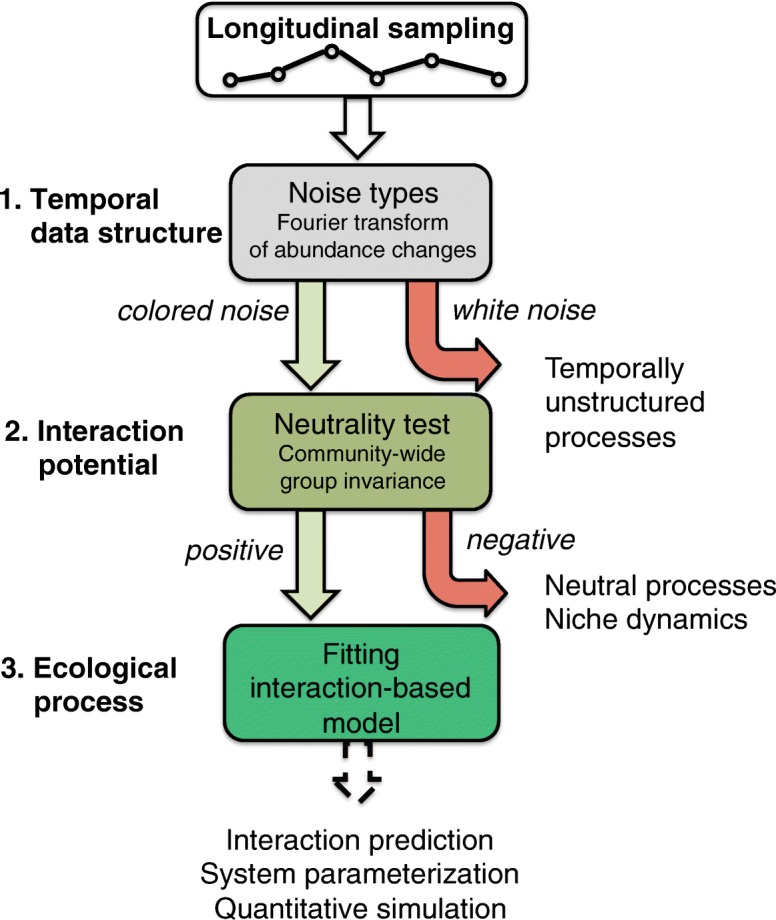
Classification of time series data. First, noise-type distributions are established. Darker noise colors indicate increasing temporal dependence between time points. White noise suggests random processes without temporal structure that can be caused for instance by technical bias such as insufficient sampling density or too large measurement noise, or reflect an intrinsic lack of time structure. Second, the presence of neutral dynamics are tested. A positive test result (*p* value below the significance level) suggests a potential for interactions in the microbial community and can be followed up for instance by fitting community models that assume interactions, by network analysis or by causal model approaches. For high levels of external noise or too large sampling intervals, the neutrality test may yield a false positive outcome. In neutral communities, variation can be suitably analyzed within stochastic frameworks

Our proposed classification scheme consists of three steps: (i) test for temporal structure, (ii) test for neutrality, (iii) fit an interaction model (Fig. 2). This classification scheme is complementary to the one proposed by Gibbons and colleagues, which does not test for the presence of temporal structure or neutrality [29]. In the following, we will apply this scheme to simulated and real-world microbial time series data.

## Results

In order to demonstrate the efficacy of our classification scheme, we generated time series with the Dirichlet-multinomial (DM) distribution, Hubbell’s neutral model, the SOI model, the Ricker model with varying levels of noise, and the generalized Lotka-Volterra model. In addition, we included time series from two individuals (A and B) from a long-term study of the human gut microbiota [3]. In total, we generated 60 community time series with various parameter settings, each including 100 species followed over 3000 time points. Since such long time series are not yet available in practice, we also repeated every test for the first 100 time points of each time series. An overview of the parameter settings, time series properties, and test results is given in Additional file 1: Table S1. Time series generation is summarized in “Methods”; model details are provided in Additional file 2.

### Noise types differentiate between unstructured and structured models

The first step of the classification scheme distinguishes between the presence of temporal structure, i.e., rules that govern the dynamics, and its absence. To test for the presence of temporal structure, we exploit the fact that it will introduce a dependency between time points that is absent otherwise. Different measures exist that can detect dependency on previous community states, such as autocorrelation, the Hurst exponent, and noise types. We focus on the latter here, but also tested the two other measures with similar results (see Additional file 3: Figure S1). The noise type of a species is obtained by decomposing its abundance fluctuations (or relative abundance fluctuations in most cases) into spectral densities at specific frequencies using a Fourier transform. When spectral densities tend to be high at low frequencies (i.e., long intervals) and vice versa, they signal a strong dependency on previous time points, which is visible as a negative slope of densities versus frequencies in the periodogram. Depending on the value of this slope in log-log scale, one can distinguish black (below − 2), brown (around − 2), pink (around − 1), and white noise (no negative slope). A shift in the noise color from black to pink indicates a reduced dependency on previous community states. In brief, the dependency on previous time points is strongest for black noise and weakest for pink noise, while it is absent for white noise.

**Fig. 3:**
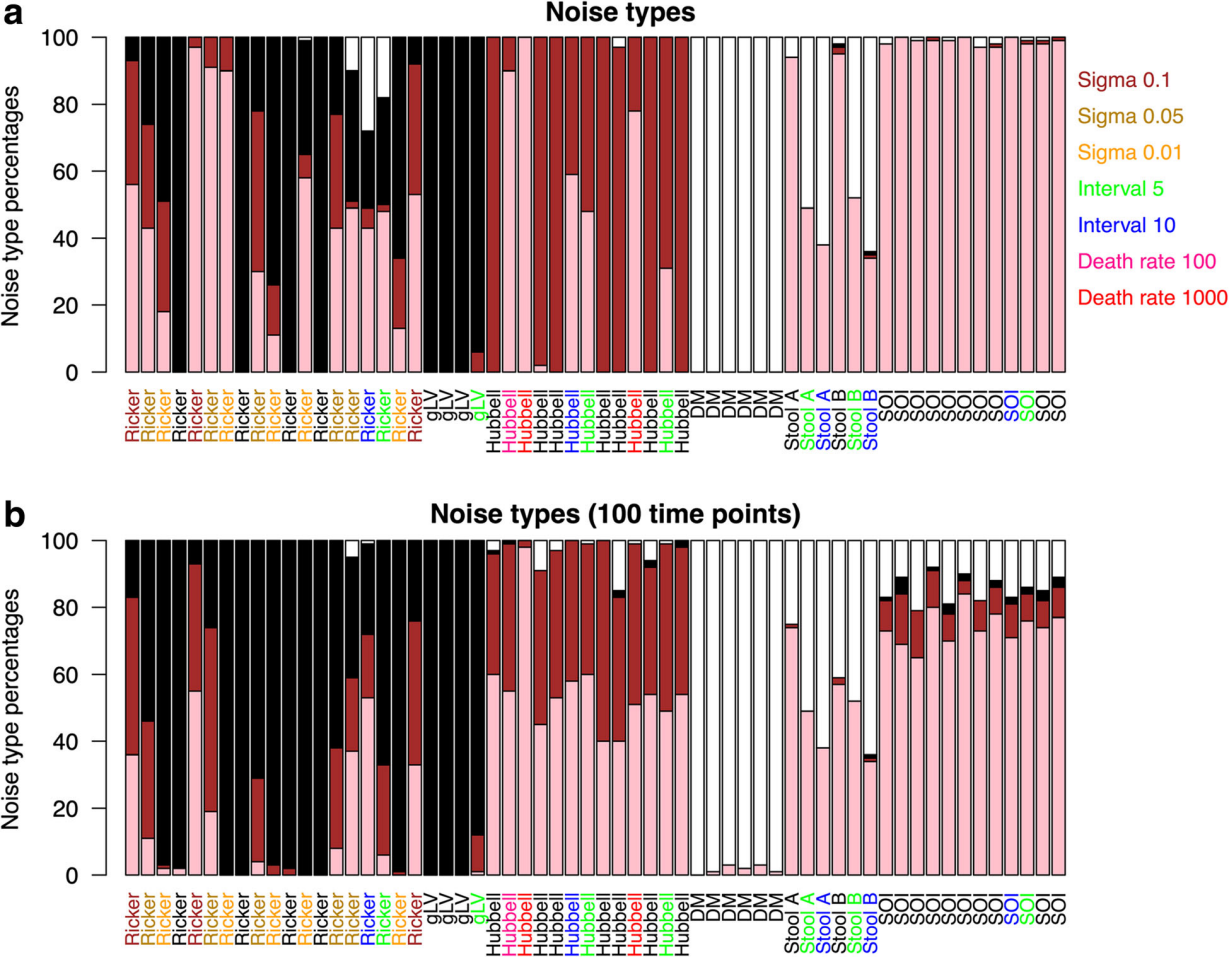
The noise-type profile distinguishes between temporally structured and unstructured community time series. **a** Noise types. **b** Noise types (100 time points). The bar plots depict for each community time series the percentage of species with white, pink, brown, or black noise. White noise indicates the absence of structure, all other noise types its presence. Labels for time series are colored according to the level of non-zero intrinsic noise (sigma) for Ricker, according to the death rate if larger than one for Hubbell, according to the interval if larger than one (with interval coloring taking precedence over sigma) and black otherwise time series for the full-length time series (**a**) as well as for shortened time series consisting of the first 100 time points **(b**)

We computed the noise type for each species and reported the percentage of black, brown, pink, and white species in each community time series (Fig. 3). While structured community models (gLV, Ricker, Hubbell, and SOI) generate time series with no or only a small percentage of white noise species, the DM distribution gives rise to mostly white noise species, as expected for unstructured data. Deterministic models such as gLV and Ricker with low intrinsic noise are dominated by black noise species, whereas Hubbell time series are mostly composed of brown noise species and SOI time series of pink noise species. However, we found that longer intervals and shorter time series increase the number of white noise species in the stool data and SOI time series, that higher connectance increases the proportion of black taxa at the cost of pink ones in Ricker but not SOI time series (Additional file 4: Figure S2 and Additional file 5: Figure S3), that high mortality rates favors pink instead of brown noise in the Hubbell time series (Additional file 6: Figure S4), and that increasing intrinsic noise increases the percentage of pink and brown noise in Ricker time series. These delicate shifts in noise-type profiles illustrate the effect of confounding factors such as sampling interval and noise. While confounders complicate recognizing an underlying model on the basis of its noise-type profile alone, they do not affect the distinction between temporally structured and unstructured community models.

This noise-type-based test for structure is also robust to compositionality and Poisson and multinomial noise (Additional file 7: Figure S5 and Additional file 8: Figure S6) and increases in accuracy with increasing number of time points (Additional file 9: Figure S7 and Additional file 10: Figure S8). While it is more effective for the first 100 time points in the transient part of the time series, it also distinguishes temporally structured from unstructured data in the last 100 time points (Additional file 7: Figure S5).

### Neutrality test distinguishes neutral from non-neutral dynamics

**Fig. 4:**
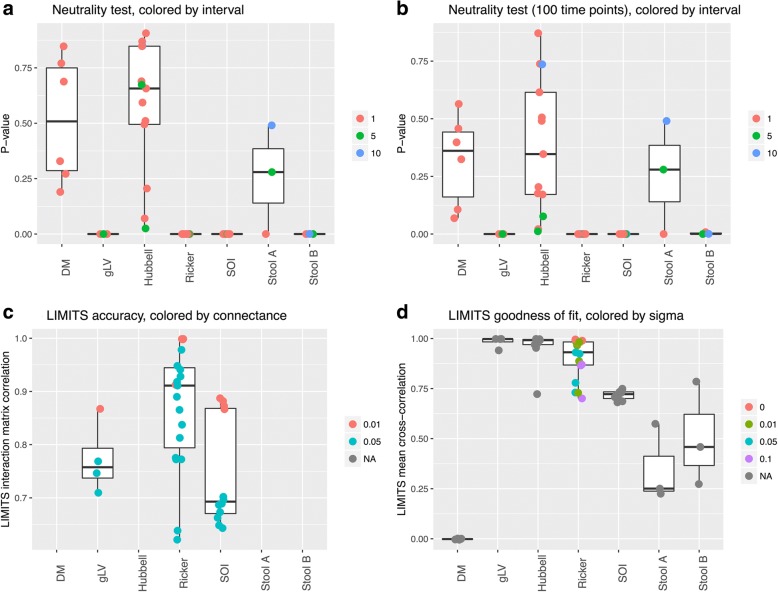
Neutrality test and LIMITS results. **a** The neutrality test distinguishes non-neutral from neutral time series. A long interval is a confounding factor. **b** The neutrality test also classifies short time series correctly. **c** The accuracy of the interaction matrix inferred with LIMITS is high for time series from the three deterministic models, but decreases with connectance. **d** The goodness of fit of time series to the Ricker model is high for all time series except for the unstructured DM data. The goodness of fit was quantified as the correlation between the original time series and the time series predicted with the Ricker model parameterized with LIMITS. Plots were made with ggplot2 [52]. The dashed lines in **a** and **b** indicate the value corresponding to a *p* value of 0.05. Values above represent significant *p* values, for which neutrality is rejected

To distinguish neutral from non-neutral structured models, we applied a test previously developed to recognize neutrality in time series data [30]. Neutrality, in this test, is defined as a general feature in which demographic rates and performance are independent of species identities. The idea of the neutrality test is to test for group invariance, which means that time series properties are not affected by summing species into groups that represent higher-level taxa such as genera or families. In particular, the group invariance of inter-species quadratic covariation is tested; thus, not only group invariance of covariance is tested, but also its correspondence to a type of covariance common to all neutral processes. When validating this test on our community time series, the DM and Hubbell time series are classified as neutral (*p* values > 0.05) as expected, whereas the other simulated time series are correctly classified as non-neutral (Fig. 4a, b). The time interval between samples is an important variable when defining neutrality, since for stool data, time series sampled at larger intervals are classified as neutral. Thus, neutrality is defined relative to the time-scale of an investigation. Furthermore, the addition of Poisson noise introduces false positives (time series falsely classified as non-neutral; Additional file 11: Figure S9).

### Microbial network inference through deterministic model fitting

The first two tests classify the stool data as resulting from a temporally structured, non-neutral community. Thus, gut microbial community dynamics is to a large part shaped by interactions between community members, which is in agreement with our knowledge of the numerous cross-feeding and competition interactions taking place between gut organisms [31]. A number of algorithms have been developed to parameterize the gLV or Ricker model directly from time series data [14, 18, 32], thereby inferring ecological interactions. Here, we employ LIMITS [14] to test this parameterization step. We applied it to the top 60 most abundant species in our test time series and measured inference accuracy as the mean correlation between the known and the inferred interaction matrix. The LIMITS algorithm was found to perform well on time series data with known interaction matrices (Fig. 4c). Interestingly, the accuracy of LIMITS is also relatively high for SOI time series, although LIMITS assumes a different community model. These results were reproduced with lower accuracy for short time series and selected time series with Poisson noise (Additional file 11: Figure S9 and Additional file 12: Figure S10).

The goodness of fit to the Ricker model was computed as the mean correlation between the community time series predicted with the parameterized Ricker in a step-wise manner and the test time series (Fig. 4d). Not surprisingly, the goodness of fit is inversely correlated to the strength of the intrinsic noise in Ricker (Spearman’s rho − 0.76, *p* value < 0.0001). We also applied LIMITS to neutral time series. Although the Hubbell model does not assume direct ecological interactions between species, it does introduce competition between all species. Notably, the goodness of fit of Hubbell time series to the Ricker model was high, but the absolute interaction strengths were significantly smaller than those inferred for all other time series, even when randomly sub-sampling from the latter to account for different sample numbers (Wilcoxon rank sum test *p* value < 0.0001).

**Fig. 5:**
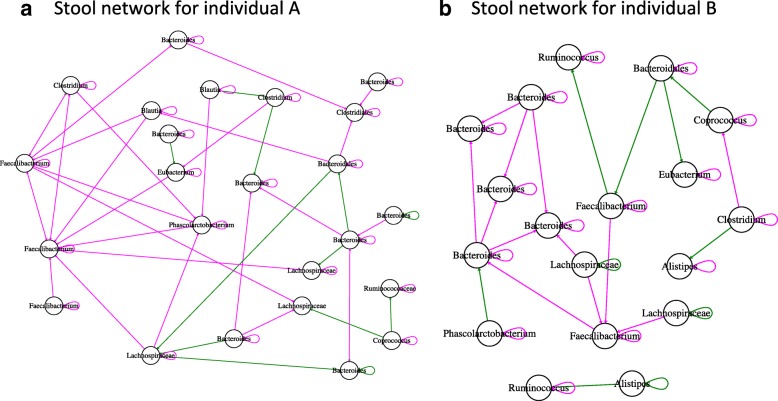
Inferred interactions in stool data sets. The inferred interaction matrices are represented as directed networks, where nodes are OTUs labeled with their genus or higher-level taxon name and directed edges represent non-zero entries in the inferred interaction matrix. Directed edges with positive signs are colored in green, those with negative in red. Orphan nodes are not shown. **a** In the network inferred for the stool time series of individual A, a negative hub is formed by a Faecalibacterium OTU. **b** In the network of individual B, the same Faecalibacterium OTU (OTU_165924) also forms a negative hub. Interaction matrices inferred with LIMITS were visualized with igraph [53]

After having tested its performance, we applied LIMITS to explore the interaction networks of the two stool time series. While gLV/Ricker models have been parameterized on stool time series previously [14, 17], the LIMITS algorithm has to our knowledge not yet been applied to the stool time series collected by David and colleagues [3] (see Additional file 13: Figure S11 for a time series plot). We noticed that repeated rarefactions alter the noise-type classification of a few taxa (Additional file 14: Figure S12) and therefore inferred interaction networks from the 30 top-abundant OTUs consistently classified as pink, brown, or black across several rarefactions. The resulting interaction networks have a large percentage of negative links (70% for individual A, 63% for individual B), in agreement with the theoretical expectation that a high percentage of negative interactions is needed to stabilize the community [33]. The same Faecalibacterium OTU (OTU_165924) forms negative hubs in both networks (with nine and five negative links, respectively; Fig. 5). In both networks, Firmicutes and Bacteroidetes OTUs form significantly more links within their phylum than expected at random, with the majority of within-phylum links being negative, while inter-phylum link numbers are not higher than expected at random. Thus, for top-abundant representatives of Bacteroidetes and Firmicutes, intra-phylum competition may be more intense than competition with members of other phyla. Our finding that Faecalibacterium forms hub nodes is in agreement with the results of an alternative network inference method (Granger causality) applied to the same data [29]. However, not all links represent ecological interactions, since networks inferred from real-world data likely contain more false positive links due to underlying environmental variables.

## Discussion

Our results show that several criteria can be combined to discriminate processes underlying the dynamics of microbial communities from time series.

The tendency of ecological time series to display pink, brown, or black noise was noticed previously [34] and linked to the environment [35]. However, since none of our tested models takes environmental factors into account, our results indicate that such noise types can result from the intrinsic community dynamics alone. Self-organized critical systems, to which the SOI model is closely related, in particular are known for their pink (1/f) noise [36], whereas the neutral model was stated previously to generate brown noise [37]. The noise types measure dependency between time points, which can be referred to as a memory effect. The strongest memory effect is found in the gLV and noise-free Ricker time series with their black noise. In general, the gLV time series quickly reach a steady state and thus represent the extreme case of perfect memory. However, for two of the noise-free Ricker time series, all taxa are classified as black while displaying periodicities. Adding a noise term in the Ricker model weakens the memory effect, thereby shifting the noise type from black via brown to pink.

It is current practice in microbial network inference to filter out rare taxa, where rareness of a taxon is defined arbitrarily by applying prevalence or abundance thresholds. Filtering taxa by noise type may constitute an interesting alternative. Taxon abundances that are not dependent on previous abundances belong to taxa that either do not interact or are too rare to reveal interactions and will thus introduce false interactions in the network model. While our work is a first step in this direction, a number of challenges have to be overcome: first, differentiating white from non-white taxa involves a threshold choice and is affected by confounding factors such as noise. For instance, we found that while overall noise-type percentages were conserved, a few (abundant) taxa in the stool data changed their noise type upon re-rarefaction. Second, it is conceivable that although white taxa are not affected by other taxa, they may influence other taxa, for instance through cross-feeding. Extensions of the gLV have been proposed previously to deal with external factors such as antibiotic treatment [14, 17] and environmental factors [4]. White taxa suspected to affect other taxa could be treated as external factors in the gLV parameterization. Tools such as MDSINE allow such a treatment directly, whereas we modified the LIMITS algorithm to support external factors. Cross-sectional microbial network inference on the time series directly or regression on the residuals after gLV model fitting may be able to pinpoint such white taxa.

In this context, we would like to emphasize that the absence of temporal structure in time series only has implications for analyses that assume its presence, such as the inference of interaction networks. Analyses that do not rely on the presence of temporal structure, such as ordination or the comparison of microbial composition between conditions, are not concerned.

In our evaluation of LIMITS, we found high correlation between inferred and known (simulated) interaction matrices. However, in real-world applications, one does not know the true interaction matrix for assessment. Instead, the observed time series is usually compared to the predicted time series [17]. Based on this criterion, LIMITS performed well on Hubbell time series, although their generating model assumes the absence of specific ecological interactions (the replacement step introduces a generic competition between all species). In general, the goodness of fit criterion is biased by the amount of memory present in the time series, since it is easier to predict a time series that is highly autocorrelated than one less so. This strongly highlights the need to test for non-neutrality before network inference. The fact that an agreement between observed and predicted time series can be misleading was also discussed in [38].

Interestingly, in our tests, LIMITS reached reasonable accuracies (quantified as the correlation between known and inferred interaction matrix) not only for Ricker but also for gLV and SOI time series. Thus, the inference is robust with respect to discrete or continuous implementation of a model (Ricker versus generalized Lotka-Volterra) and even to the resolution (populations in the Ricker and gLV model, individuals in the SOI model). In addition, LIMITS performance was not much affected by Poisson noise. However, as also pointed out by Cao et al. [39], network inference accuracy drops with an increasing sampling interval.

The first step in our proposed classification scheme, i.e., the computation of noise types, is robust to compositionality, the presence or absence of transient dynamics, the presence of Poisson and multinomial noise and to the tested sampling intervals and works for relatively short time series, yet it is currently unknown how sensitive it is to the impact of the environment. While the environment may influence community dynamics [40], we may reasonably assume that it will only in extreme cases introduce temporal structure when it is absent otherwise. Moreover, as we remove the linear trend from the time series prior to power spectrum estimation, our approach should be robust to slowly varying environmental conditions. However, this point requires further investigation. It is of note that all the models with temporal structure presented in this work can be modified to account for the influence of external drivers. Although a number of confounding factors (strength of intrinsic noise, external noise, sampling interval) can alter the noise type and hence prevent the identification of the underlying model from the noise-type profile, these processes do not introduce non-white taxa when these are absent. Thus, the presence of non-white taxa is a robust indicator of temporal structure.

The second step, which tests for neutrality, is more sensitive to sampling interval and external noise than the first. While a high percentage of brown noise taxa gives an additional and independent hint for neutral dynamics, this indicator can also be biased by the effects of intrinsic noise, a high death rate and the sampling interval. This highlights the importance of sufficiently dense sampling in longitudinal studies.

According to our tests, the stool data are temporally structured and non-neutral, the latter in agreement with previous results [19, 41]. The noise-type profile of the stool data is closest to the noisy Ricker and SOI time series, indicating the presence of stochasticity. This overall deterministic behavior with a stochastic component agrees well with our expectation for a microbial community whose members are known to engage in various cross-feeding and competitive interactions while being exposed to daily perturbations through the host (e.g., diet).

## Conclusion

In conclusion, we have demonstrated that knowledge about the fundamental properties of microbial community dynamics is required for unbiased community study and model inference. We have proposed a classification scheme for microbial sequencing time series that first differentiates between unstructured and structured models, then tests for neutrality and finally determines the goodness of fit to a deterministic model. To make these tests accessible, we have implemented a new R package called seqtime. While several hurdles still have to be overcome, this classification scheme is a first step towards model identification from time series data.

## Methods

### Simulations and empirical data

Below, data generation is briefly described. More detailed model descriptions can be found in Additional file 2, while model parameters are given in Additional file 1: Table S1.

#### Neutral (Hubbell) model

We initialized the local community with 10 species of even proportions and omitted the first 1000 time steps, except for the immigration rate of 0.1, where we omitted the first 5000 time steps due to the slower convergence of the dynamics with low immigration rates. The metacommunity species proportions were set to the initial species proportions. The speciation rate in the metacommunity is zero; hence, the metacommunity composition is constant.

We generated all neutral test time series with the simHubbell function in the seqtime R package. As a control, we also computed noise types for time series generated with the untb [42] and the WrightFisher R packages [30].

#### Interaction matrix generation

The SOI, Ricker, and gLV models take an interaction matrix as a parameter, which specifies which species interacts with which other species.

We used the algorithm by Klemm and Eguíluz [43] to generate modular and scale-free interaction matrices that reproduce properties of inferred microbial networks [44, 45]. We set the clique number parameter of the Klemm and Eguíluz algorithm to 10.

We assigned interaction strengths by setting diagonal values to − 1 and sampling off-diagonal values from a uniform distribution between 0 and 1. We then adjusted interaction matrix connectance (the ratio of non-zero to all values in the interaction matrix omitting the diagonal) to 0.05 or 0.01, which is close to the range reported for food webs [46] and within the range of inferred microbial networks [47].

Interaction matrices need to contain a large number of negative interactions to avoid unbounded increase of species abundances [33, 48, 49]. We therefore converted randomly selected positive interactions into negative ones. After each conversion, we tested matrix stability with a Ricker simulation and stopped once a stable matrix was obtained. In this way, we generated interaction matrices with a positive edge percentage of 0, 16, 40, and 64%.

#### Generalized Lotka-Volterra (gLV) model

The gLV model describes community dynamics as a function of growth rates and species interactions. We generated the interaction matrix as described above and sampled the growth rates from a uniform distribution with values between 0 and 0.5.

#### Ricker model

The Ricker model is a discrete version of the gLV model. In addition to the interaction matrix, it takes a vector of carrying capacities as input. We generated the carrying capacities from a uniform distribution with values between 0 and 0.5. As suggested by Fisher and Mehta [14], we also include a noise term with strength *σ*.

#### SOI model

The SOI model (based on model B, [22]) is individual-based and takes into account species-specific immigration and extinction probabilities as well as asymmetric interactions between individuals. We set the immigration rates to the initial species proportions (described below) and generated extinction rates from a uniform distribution between 0 and 1.

#### Dirichlet-multinomial distribution

The DM distribution takes two parameters, namely the species proportion vector (set to the initial species proportions) and the overdispersion parameter *θ*, set to 0.2, 0.02, or 0.002. These overdispersion values have been reported for sequencing data previously [47].

#### Time series simulations

With each model, we simulated the dynamics of 100 species for 3000 time steps. We generated initial species proportions with the broken stick process [50] implemented in vegan’s function bstick. We also generated test time series with even initial species proportions.

We tested three sampling rates: once every time step, once every 5 time steps, and once every 10 time steps.

#### Stool time series data

The stool data consist of two metagenomic time series of fecal samples that were collected almost daily by two individuals [3]. We rarefied the counts to 10,000 reads per sample and omitted the last time point from individual B, since there was a gap of 66 days between it and the previous sample. We then interpolated the data with function stineman in the stinepack R package [51] to ensure equidistant time intervals. A few small negative values introduced by the interpolation were set to zero. After interpolation, the data set from individual A included 365 time points and the data set from individual B 253 time points. Finally, we selected the 100 top-abundant OTUs, ranked by their sum across time points.

#### Poisson noise

We scaled gLV and Ricker time series by a factor of 1000, Hubbell time series by a factor of 2, DM data by a factor of 1, and SOI time series by a factor of 50 to obtain counts for gLV and Ricker and similar sequencing depths across models. We then generated noisy time series according to the formula: *y*_*ij*_ = Pois(*x*_*ij*_), where *x*_*ij*_ is the count of the *i*th species in the *j*th sample and *y*_*ij*_ is the Poisson-distributed value. We applied LIMITS to selected noisy time series including 12 Ricker, 12 Hubbell, and 12 SOI time series.

#### Multinomial noise

Noise was generated by applying the multinomial distribution to the taxon proportions in each sample. The sequencing depth was varied randomly between 1000 and 1500 with a uniform distribution. Data were converted into relative abundances before noise-type classification.

### Computation of time series properties

All properties were computed for the full-length time series as well as for the first 100 time points. Raw abundances were converted into relative abundances.

#### Noise types

Frequency and spectral density are calculated for each species with R function spectrum with detrending enabled. Detrending removes linear trends by computing the residuals of the least-squares fit of a line. In log-log scale, a slope of − 1 indicates pink (1/f) noise, a slope of − 2 brown noise, and a slope below black noise, whereas white noise is characterized by a slope around 0. We determine the slope by first fitting a spline with function smooth.spline (whose degree of freedom is set to the maximum of [2,log10(length(time series))]) and then computing the minimum of the first derivative of the spline. In this way, we can accommodate to an extent non-linear relationships between frequency and spectral density, where the amount of non-linearity allowed depends on the length of the time series. We then classify a species as black when the slope is below − 2.25, as brown when it is in the range of (− 1.75, − 2.25], as pink when in the range of (− 0.5, −1.75], and as white otherwise. These boundaries avoid unclassified species. However, since the boundaries are arbitrarily chosen, we also tested a more stringent definition with an allowed deviation of ± 0.2 from − 1 for pink and from − 2 for brown noise, which introduced unclassified species, but did not affect our conclusions (data not shown).

#### Maximal autocorrelation and Hurst exponent

For each species, we computed the maximal autocorrelation for lags larger than 0 with R function acf and the Hurst exponent with function HurstK in R package FGN. We assigned species to four arbitrarily selected maximal autocorrelation bins (< 0.3, [0.3,0.6), [0.6,0.95), > 0.95) and Hurst exponent bins (< 0.6, [0.6,0.8),[0.8,0.9), > 0.9) and computed the percentage of species in each bin.

### Neutrality test and LIMITS

#### Neutrality test

The neutrality test [30] tests the per-capita equivalence of species by determining whether or not the covariances between species are invariant to grouping. The test relies on a constant-volatility transformation that stabilizes the volatility of a two-group (two-species) neutral community irrespective of how species are grouped. Neutrality was tested on relative abundances using 500 randomly drawn constant-volatility transformations through the function NeutralCovTest in the WrightFisher R package (https://github.com/reptalex/WrightFisher) with method logitnorm. *p* values produced from the neutral covariance test were used as a measure of the incompatibility of the data with the neutral model.

#### LIMITS

We translated the LIMITS algorithm [14], originally implemented in Mathematica, into R. We then ran LIMITS on the 60 top-abundant species of relative abundance time series. When the inferred interaction matrix had at least one eigenvalue with a positive real part, we applied a Schur decomposition and modified the diagonal part to avoid explosions when predicting time series. We assessed the accuracy by computing the mean correlation of the known and the inferred interaction matrix rows and the goodness of fit as the mean correlation of the observed and predicted community time series. The predicted time series was computed with the parameterized Ricker model in a step-wise manner, i.e. the values at each time point are computed from the original values at the preceding time point using the predicted interaction matrix. The carrying capacity of a species was estimated as the mean of its abundance across time points.

#### Network analysis

Links between phyla were counted as the number of entries in the interaction matrix, including the diagonal. The significance of intra- and inter-phylum link number was assessed by repeatedly (100 times) randomizing the interaction matrix while preserving the total number of entries and computing parameter-free *p* values.

## Additional files

Additional file 1: **Table S1.** lists for each test time series the parameters used to generate it (sheet “Model parameters”) and the properties of relative abundance time series, which include noise type, autocorrelation, Hurst bin percentages, the *p* values of the neutrality test, and the LIMITS results (sheet “Time series properties”). In addition, it includes all these results for the first 100 time points (sheet “First 100 tp properties”), the last 100 time points (sheet “Last 100 tp properties”) and for time series with Poisson noise (sheet “Poisson time series properties”). (XLSX 158 kb)

Additional file 2: Ecological models. (DOCX 1011 kb)

Additional file 3: **Figure S1.** Maximal autocorrelation and Hurst exponent profiles reproduce patterns seen with noise types. (a) The species in each time series are grouped in four bins according to their maximum (lagged) autocorrelation (white: below 0.3, light blue: 0.3 to 0.6, blue: 0.6 to 0.95, dark blue: above 0.95). (b) The species are separated into four Hurst exponent bins, ranging from white (below 0.6), orange (0.6 to 0.8), red (0.8 to 0.9) to dark red (above 0.9). Species for which the maximum autocorrelation or Hurst exponent could not be computed (due to a large number of zeros) are colored in gray. Labels for time series are colored according to the level of non-zero intrinsic noise (sigma) for Ricker, according to the death rate if larger than one for Hubbell, according to the interval if larger than one (with interval coloring taking precedence over sigma) and black otherwise. (PDF 9 kb)

Additional file 4: **Figure S2.** The noise-type classification and the neutrality test for Ricker and gLV are robust to positive edge percentage, but connectance affects noise types in Ricker. (a, c) The percentage of taxa with black, brown, pink and white noise types is plotted against the connectance of the interaction matrix for Ricker and gLV, respectively. The percentage of black taxa in Ricker was positively correlated to connectance (Spearman’s rho: 0.86, *p* value < 0.00001), whereas the percentage of pink taxa in Ricker was negatively correlated to connectance (Spearman’s rho: − 0.71, *p* value = 0.00049). (b, d) The percentage of taxa with black, brown, pink, and white noise types is plotted against the positive edge percentage of the interaction matrix for Ricker and gLV, respectively. All neutrality test *p* values were zero, indicating non-neutral dynamics. Time series were generated for 100 species and 3000 time points. (PDF 16 kb)

Additional file 5: **Figure S3.** The noise-type classification and the neutrality test for SOI are robust to interaction matrix properties. (a) The percentage of taxa with black, brown, pink, and white noise types is plotted against the connectance of the interaction matrix. (b) The percentage of taxa with black, brown, pink and white noise types is plotted against the positive edge percentage of the interaction matrix. The stochasticity in the SOI model is plotted against (c) noise types and (d) neutrality *p* values. Stochasticity is defined here as the ratio between the mean of extinctions and immigrations and the mean of the absolute interaction strengths (excluding diagonal values). Neutrality test *p* values for (a) and (b) were zero, indicating non-neutral dynamics. Time series were generated for 100 species and 3000 time points. (PDF 16 kb)

Additional file 6: **Figure S4.** The noise-type classification and the neutrality test are robust for a wide parameter range in the Hubbell model, but noise types are affected by the death rate. (a) The percentage of taxa with black, brown, pink and white noise types is plotted against the death rate. There is a significant negative correlation between the percentage of brown species and the death rate (Spearman’s rho: − 0.85, *p* value < 0.000001) and a corresponding positive correlation of the percentage of pink species to the death rate (Spearman’s rho: 0.94, *p* value < 0.000001). (b) The *p* values of the neutrality test are plotted against the death rate. (c) The percentage of taxa with black, brown, pink, and white noise types is plotted against the number of individuals. (d) The *p* values of the neutrality test are plotted against the number of individuals. (d) The percentage of taxa with black, brown, pink, and white noise types is plotted against the immigration rate. (e) The *p* values of the neutrality test are plotted against the immigration rate. Neutrality is rejected for a *p* value below 0.05. The *p* value of 0.05 is indicated by a dashed horizontal line. Time series were generated for 100 species and 3000 time points. For the immigration rate, the percentage of noise types of taxa with non-zero abundances was plotted, since for the low immigration rates tested in this simulation, many taxa have abundances of zero. (PDF 40 kb)

Additional file 7: **Figure S5.** The test for temporal structure with noise types is robust to compositionality and the absence of transient dynamics. (a) The noise-type profiles for absolute abundances do not differ noticeably from those for relative abundances shown in Figure 3a. (b) When noise types are computed for the last hundred time points, most time series are correctly classified as temporally structured or unstructured. Labels for time series are colored according to the level of non-zero intrinsic noise (sigma) for Ricker, according to the death rate if larger than one for Hubbell, according to the interval if larger than one (with interval coloring taking precedence over sigma) and black otherwise. (PDF 8 kb)

Additional file 8: **Figure S6.** The test for temporal structure with noise types is robust to noise. (a) Noise-type profile in the presence of noise generated with the Poisson distribution for each species and each sample. (b) Noise-type distribution in the presence of noise generated with the multinomial distribution for each sample. Labels for time series are colored according to the level of non-zero intrinsic noise (sigma) for Ricker, according to the death rate if larger than one for Hubbell, according to the interval if larger than one (with interval coloring taking precedence over sigma) and black otherwise. (PDF 8 kb)

Additional file 9: **Figure S7.** Increasing the time series length improves the accuracy of the test for temporal structure. Noise types were computed for time series sub-sets from 1000 to 1010 (a) and 1000 to 1025 (b) for all data sets with more than 1000 time points. Labels for time series are colored according to the level of non-zero intrinsic noise (sigma) for Ricker, according to the death rate if larger than one for Hubbell, according to the interval if larger than one (with interval coloring taking precedence over sigma) and black otherwise. (PDF 8 kb)

Additional file 10: **Figure S8.** Increasing the time series length improves the accuracy of the test for temporal structure. Noise types were computed for time series sub-sets from 1000 to 1050 (a) and 1000 to 1100 (b) for all data sets with more than 1000 time points. Labels for time series are colored according to the level of non-zero intrinsic noise (sigma) for Ricker, according to the death rate if larger than one for Hubbell, according to the interval if larger than one (with interval coloring taking precedence over sigma) and black otherwise. (PDF 7 kb)

Additional file 11: **Figure S9.** The presence of noise decreases the accuracy of the neutrality test but affects network inference accuracy less. (a) For the last 100 time points, when many simulated time series reach equilibrium, neutrality is erroneously rejected for several Hubbell time series and erroneously detected for a number of Ricker and SOI time series. The classification does not change for the stool time series. (b) The addition of Poisson noise does not introduce false negatives in the neutrality test, but introduces false positives (i.e., Hubbell time series for which neutrality is rejected). The dashed lines in (a) and (b) indicate the value corresponding to a *p* value of 0.05. For values above, neutrality is rejected. (c) LIMITS accuracy, i.e., mean correlation of inferred and known interaction matrix, for time series with Poisson noise. Inference failed for gLV time series. (d) LIMITS goodness of fit for time series with Poisson noise. The goodness of fit was computed as the mean correlation between original and predicted time series. The data points are colored according to the interval in panels (a), (b) and (d), and according to the connectance in panel (c). (PDF 17 kb)

Additional file 12: **Figure S10.** The accuracy of network inference with LIMITS decreases more strongly when applied to the last 100 than to the first 100 time points. (a) LIMITS accuracy, i.e., mean correlation of inferred and known interaction matrix, for the first 100 time points. (b) LIMITS goodness of fit for the first 100 time points. The goodness of fit was computed as the mean correlation between original and predicted time series. (c) LIMITS accuracy for the last 100 time points. Since gLV time series are constant, no network could be inferred for them. (d) LIMITS goodness of fit for the last 100 time points. The correlation between the goodness of fit to the Ricker model and the intrinsic noise strength observed in noise-free time series is lost. The data points are colored according to the connectance in panels (a) and (c), according to interval in panel (b) and according to the intrinsic noise strength sigma in panel (d). (PDF 17 kb)

Additional file 13: **Figure S11.** Time series of the 100 top abundant OTUs in the processed stool data of individual A and B [3]. The OTUs are colored according to their noise type (with cyan for white noise). (PDF 17 kb) (PDF 219 kb)

Additional file 14: **Figure S12.** Variability of noise-type classification across rarefactions. The noise types of 100 taxa selected to be top abundant in one rarefaction were computed for repeated rarefactions in the stool data set of individual A [3]. (PDF 5 kb)
