# Supplementary material for: Signatures of ecological processes in microbial community time series
Source: Microbiome. 2018 Jun 28;6:120. doi: 10.1186/s40168-018-0496-2 (PMC6022718; doi:10.1186/s40168-018-0496-2)
Supplement: Supplementary file 2 — Ecological models. (DOCX 1011 kb) [file 40168_2018_496_MOESM2_ESM.docx]

**Ecological models**

1. **Model hypotheses**

The four models considered in this paper, namely the generalized Lotka-Volterra (gLV), Ricker, Hubbell and Self-Organized Instable (SOI) models, are built on standard ecological concepts that shape ecosystem structure (Solé & Bascompte 2006). These are environment limitations and fluctuations, immigration, extinction and interactions. For each of these concepts, we recall its definition or meaning and discuss its implementation (or absence) in the four models. The resulting hypotheses are summarized in Table A.

1. **Environment limitation and fluctuation**

*Carrying capacity and equilibrium.* Following (Hui 2006), for a given environment (defined in terms of nutrient availability, temperature, pH, humidity, etc.) we define its carrying capacity as its maximal persistently supportable population load. The population steady state is the long term stationary species abundance distribution, which, due to mortality or species interactions, does not necessarily correspond to a saturation of the carrying capacity. It is thus important to distinguish both notions.

In the Hubbell and SOI models, the system size is fixed by the maximum number of individuals that it can support. Note that here, individual has to be taken in a broad sense, and may represent a patch of microbes from the same species. These models thus take explicitly into account a global, constant carrying capacity of the environment (i.e. the grid size, see II.4). The Hubbell model assumes that the environment is always saturated, whereas this is not the case for the SOI model, in which the total ecosystem population can be less than the environment carrying capacity (see Figure A below). Note that in both models, each individual occupies the same fraction of the carrying capacity, regardless of its species. Consequently, species' absolute abundances can be transformed into percentages of the environment carrying capacity. In the Ricker model, there is no built-in representation of the environment carrying capacity. However, the model takes explicitly into account the existence of a steady state in the abundance distribution. Since these stationary abundances are computed as time averages of each species abundance, the total abundance in simulations with the Ricker model fluctuates around a stable equilibrium distribution. Finally, the gLV model does not explicitly specify the global carrying capacity or a stable steady state distribution. A species-specific carrying capacity is however implicitly specified through the model parameters. The existence of the steady state depends on parameter values. We have selected parameter values that lead to stable steady states (no explosion of species abundances). It is also known that gLV may occasionally display complex dynamics such as limit cycles or chaos, but we never observe these cases in our simulations (presumably because they occur for specific parameter values).

Figure A: The differences in the implementation of carrying capacity are illustrated with time series of the total abundance in the four models, with experiment identifiers 39, 1, 14 and 37 respectively (absolute abundances). The red line indicates the expected carrying capacity (number of individuals specified in the Hubbell and SOI simulations).

*Stochastic fluctuations.* Stochastic individual behaviour (Hubbell and SOI models) or overall population growth rate (Ricker model with noise, see II.2) may account for intrinsic individual stochasticity or random fluctuations in the environment. In Hubbell and SOI models, the level of noise is determined by the grid size (maximal number of individuals). The gLV model is deterministic and does not account for intrinsic or environmental fluctuations.

1. **Immigration and intrinsic growth**

Immigration assumes that there is an inflow of individuals in the ecosystem coming from an external pool of species. The intrinsic growth rate is the rate at which an individual from a given species reproduces or disappears in the absence of any other individual, that is in the absence of intra and interspecies interactions.

Hubbell’s model takes into account immigration and intrinsic growth. As the carrying capacity of the environment is always saturated, death is tightly coupled with immigration and birth (local replacement), since resident individuals that disappear are exactly replaced by immigrants or offspring of local residents, regardless of their respective species. The general SOI model accounts for immigration, death and intrinsic growth. However, the version implemented here is a slightly modified version of model B in (Solé 2002), in which intrinsic growth is not explicitly represented. It should be noted that due to the unsaturated carrying capacity, the two remaining processes, namely immigration and death, are decoupled. Ricker and gLV models do not include immigration and involve for each species a growth rate resulting from the balance between birth and death rates.

1. **Interactions**

Hubbell’s model does not model direct ecological interactions between species in the ecosystem. However, as the total population is constant, local replacement induces apparent equal indirect +/- exploitation, in which the exploiting interaction partner generates an offspring that will replace the other, between all species. In the SOI model, interactions are stochastic. In the version implemented here, two types of interactions occur: +/- exploitation, or +/0 commensalistic interactions, in which the stronger participant generates an offspring without harming the weaker.

In the Ricker and gLV models, the interactions are deterministic (but the overall growth rate resulting from intrinsic growth and interactions is perturbed by a multiplicative random term in Ricker). They may be of any type.

| **Concept or feature ->**  **Model**  **↓** | **Carrying Capacity**  **or environmental limit** | **Immigration,**  **Intrinsic growth** | **Interactions** | **Stochastic  processes** |
| --- | --- | --- | --- | --- |
| **Hubbell** | fixed system size (constant total number of individuals) | coupled death, immigration and  birth | indirect | immigration  death |
| **SOI**  **(adapted from model B in Solé 2002)** | maximal system size (maximum number of individuals) | no intrinsic birth | exploitation (+/-)  commensalism (+/0) | immigration, death and interactions |
| **Ricker** | species-specific carrying capacity defined through the steady state | no immigration | all types | overall growth rate |
| **gLV** | species-specific carrying capacity specified | no immigration | all types | no |

**Table A.** Summary of the model features.

1. **Modelling absolute or relative abundances**

Biological data available for microbial ecosystem modelling are usually expressed in terms of relative species abundance, since absolute counts are often not measured. Thus, the observed quantities are

$$r_{i}=\frac{x_{i}}{\sum_{S} x_{i}}$$

where S is the set of all species observed in the ecosystem and $x_{i}$ is the absolute abundance of species i. However, the four models describe the time evolution of absolute abundances in an ecosystem.

In the absence of any external measurement equal or proportional to the total absolute abundance $\sum_{S} x_{i}$ along the time series, the problem is usually overcome by assuming that $\sum_{S} x_{i}$ is constant or undergoes slow variations compared to the sampling time scale, so that it can be considered as locally constant in time. It should be noted that this assumption precludes the modelling of time series where the total abundance of microorganisms strongly varies with time, such as for instance colonization or resilience after an antimicrobial treatment. We will therefore focus on situations where this assumption is valid.

In practice, for complex ecosystems with many species, only the most abundant or prevalent species are modelled, since zeros in species with lower prevalence are ambiguous, reflecting either a true absence or a presence below the detection limit. This means that only a subset of the $S$ species, denoted $S’$ is retained, whose relative abundances $r_{1}$, …, $r_{S'}$ satisfy $r_{1}+r_{2}+\cdots+r_{S^{'}}<1$.

For Hubbell’s model, in which the total number of individuals is constant and species are not involved in direct interactions, using relative instead of absolute abundances is not an issue. Indeed, the constant value of the total absolute abundance is the carrying capacity of the environment and the relative abundances represent the fraction of the carrying capacity occupied by each species.

In gLV and Ricker model, it is not possible in general to generate trajectories with S species such that the total abundance is constant in time. However, if it can be considered as locally constant in time, the strict subset $S’$ of relative abundances $r_{1}$, …, $r_{S'}$ still follows a gLV or Ricker model up to the multiplication of the interaction coefficients by a positive constant, provided there are no or only very weak interactions with the neglected species (Fisher & Mehta 2014). As we are mainly interested in the signs and relative size of the coefficients, this is not a limitation.

Finally, for the SOI model, assuming a constant total abundance is not consistent with the model, since the total abundance is stochastic, as illustrated in Figure A. If in spite of this, we assume small or slow variations around a constant value (noted K), the relative abundances $r_{1}$, …, $r_{S}$ are fractions of K. They are related to the corresponding fractions $f_{1}$, …, $f_{S}$ of the total carrying capacity (noted N) by $r_{i}=(N{/K)f}_{i}$.

1. **Model description**
2. **Generalized Lotka-Volterra model**

The generalized Lotka-Volterra (gLV) model describes the continuous evolution of species abundance over time by a set of ordinary differential equations (ODE):

$$\frac{dx_{i}}{dt}=x_{i}\left( b_{i}+\sum_{j} c_{ij}x_{j} \right)$$

In these equations, *b_i_* represents the birth rate and *c_ij_* are the interaction coefficients. Note that *K_i_ = -b_i_ / c_ii_* is the carrying capacity of species *i* in absence of other species (*c_ii_* < 0). This is the steady state value ultimately reached by species *i* when growing alone. In presence of other, positively or negatively interacting species, this steady state value may be either higher or lower than *K_i_*.

The formula for the carrying capacity is obtained in the following way: in the absence of other species and assuming self-inhibition ($c_{ii}<0)$, the abundance change of species $i$ is described by the following equation:

$$\frac{dx_{i}}{dt}=x_{i}\left( b_{i}+c_{ii}x_{i} \right)$$

Since $c_{ii}$ is negative, its absolute value, denoted as $\left| c_{ii} \right|$, is such that $\left| c_{ii} \right|=-c_{ii}$

such that the equation can be expressed as

$$\frac{dx_{i}}{dt}={\left| c_{ii} \right| x}_{i}\left( \frac{b_{i}}{\left| c_{ii} \right|}-x_{i} \right)$$

Setting $K_{i}=\frac{b_{i}}{\left| c_{ii} \right|}=-\frac{b_{i}}{c_{ii}}$, the equation becomes a standard logistic growth equation

$$\frac{dx_{i}}{dt}={\left| c_{ii} \right| x}_{i}\left( K_{i}-x_{i} \right)$$

in which $K_{i}$ is the carrying capacity of species $i$.

1. **Ricker model**

The Ricker model, as described in (Fisher & Mehta 2014) is a discrete-time version of a gLV model. Let $\Delta t$ be the sampling time step (here assumed to be equal to 1 without loss of generality), we denote $t_{n}=n\Delta t$ the sampling times and $x_{i,n}$ the absolute abundance of species $i$ at time $t_{n}$, then the model equations are

$$\text{ln}\left( x_{i,n+1} \right)=\text{ln}\left( x_{i,n} \right)+\Delta t\sum_{j} c_{ij}(x_{j,n}-\bar{x}_{j})+\varepsilon_{i,n}$$

where $\bar{x}_{j}\geq0$ is the steady state abundance of species $j$, the $c_{ij}$ are interaction coefficients that describe the influence of species $j$ on the abundance of species $i$ and the $\varepsilon_{i,n}$ are i.i.d Gaussian variables with zero mean and variance equal to $\sigma^{2}\Delta t$, accounting for environment and population random fluctuations.

In the absence of noise, the abundance distribution defined by $(\bar{x}_{1},\ldots, \bar{x}_{M})$ is a steady-state solution of the model, possibly non unique if the interaction coefficient matrix is singular. Moreover, as demonstrated in (Fisher & Mehta 2014), in the limit $\Delta t\to0$, the Ricker model reduces to a specific version of the continuous time generalized Lotka-Volterra model

$$\frac{dx_{i}}{dt}=x_{i}\left( \sum_{j} c_{ij}\left( x_{j}-\bar{x}_{j} \right) \right)$$

The original LIMITS algorithm (Fisher & Mehta 2014) infers the interaction matrix of the Ricker model from time series. It can be extended to systems subject to time-dependent perturbations affecting the dynamics according to the following equations:

$$\text{ln}\left( x_{i,n+1} \right)=\text{ln}\left( x_{i,n} \right)+\Delta t\sum_{j} c_{ij}(x_{j,n}-\bar{x}_{j})+\sum_{k} \text{ϵ}_{ik}u_{k,n}+\varepsilon_{i,n} .$$

In order to estimate the interaction matrix and the strength of the susceptibilities to those perturbations, we introduce the following variables in a similar fashion as described in (Fisher & Mehta 2014):

$$Y_{in}= \text{ln}\left( x_{i,n+1} \right)-\text{ln}\left( x_{i,n} \right), C_{i\omega}=\left( c_{ij},\text{ϵ}_{ik} \right), X_{\omega n}=\left( x_{j,n}, u_{k,n} \right).$$

The essential idea of the algorithm is to infer the interaction matrix and susceptibilities summarized in the matrix $C_{i\omega}$by computing the pseudo-inverse of the matrix X:

$$C_{i\omega}^{estim}= Y_{in} . X_{n\omega}^{-1}$$

To assess the estimation error, the data is split into training and testing sets and the species are added one by one according to the error function. The procedure is applied *r* times and the final estimation is given by the median over the ensemble of estimates.

1. **Hubbell’s neutral model**

The neutral model of community dynamics assumes per-capita equivalence in the probability of birth, death, and immigration events across all individuals, regardless of their species (Washburne 2016). In our implementation, the parameters of the neutral model include species proportions in the metacommunity and in the initial local community as well as the number of individuals removed at each time step (death rate), the immigration rate and the number of individuals.

1. **SOI**

This model is a modification of model B in (Solé 2002). It is a stochastic individual-based model with discrete time-steps $\Delta t$ and perfect mixing, meaning that during one given time step, an individual microbe can potentially interact with any other microbe. The model assumes a finite grid of N available sites for microbial colonization. Each site can be occupied by exactly one individual of one microbial species from a finite pool of S species. As a consequence, 1/N can be interpreted as the fraction of the total carrying capacity devoted to one individual, and all the microbes in the ecosystem have equal impact on the environment, regardless of their species.

The model is parameterised with two nonnegative S-dimensional vectors $M=\left( \mu_{1}, \cdots\mu_{S} \right)$ and $E=\left( \varepsilon_{1}, \cdots\varepsilon_{S} \right)$ and an S x S matrix $\Omega=\left( \omega_{ij} \right)$ in order to model different stochastic events:

1. immigration : an empty site may be occupied by an individual from species $i$ at rate $\mu_{i}$, that is with probability $\mu_{i}\Delta t$
2. extinction: a site occupied by an individual from species $i$ may become empty at rate $\varepsilon_{i}$
3. exploitation: if two sites respectively occupied by an individual from species $i$ and an individual of species $j$ are such that $\omega_{ji}<0$ and $\omega_{ji}<\omega_{ij}$ then the individual of species $j$ is repaced by a new individual of species $i$, at rate $r_{ij}=\omega_{ij}-\omega_{ji}$
4. commensalism: if three sites are such that two of them are respectively occupied by an individual from species $i$ and an individual of species $j$ with $\omega_{ji}\geq0$ and $\omega_{ji}<\omega_{ij}$ and the third site is empty, then it may be occupied by a new individual of species $i$ at rate ${r_{ij}=\omega}_{ij}+\omega_{ji}$

The $\mu_{i}$’s and $\varepsilon_{i}$’s are nonnegative immigration and extinction rates. For a pair of species $i,j$ the coefficient $\omega_{ji}$ models the influence of species $i$ on species $j$ and $\omega_{ij}$ the influence of species $j$ on species $i$. They may be positive, negative or zero.

As the parameter dimension of the model is high, it is mainly used for simulation or to assess the sensitivity of the model to its parameters, in order to study global features of the ecosystem. In (Solé 2002) it is used to reproduce rank-abundance distributions in various situations.

1. **Relationships between models**

Models in ecology can be formulated at different scales.

Stochastic individual-based models (sIBM) form a popular model category in which individuals (in a broad sense, these can be actual individuals or groups or patches) behave and interact according to stochastic rules. The Hubbell and SOI models considered in this paper are both sIBM.

These models usually depend on a scale parameter $N$, representing the “size” of the model.

For instance, $N$ can be an idealization of the available space, or the typical or maximum number of individuals in the ecosystem. Denoting with $N_{i}$ the number of individuals of species $i$ in the model, the rescaled abundance is $x_{i}=\frac{N_{i}}{N}$. Depending on the interpretation of $N$, it will be a density or a frequency.

It is well known that under suitable mathematical conditions, for very large $N$ the rescaled process can be approximated by a system of stochastic differential equations (SDE), of the form

|  | $dx=f(x)dt+\frac{1}{\sqrt{N}}V(x)dW$ | (1) |
| --- | --- | --- |

Where $x=\left( x_{1},x_{2},\ldots,x_{n} \right)^{T}$ and $f$ and $V$ are functions that do not depend on $N$ and $dW$ is a Gaussian unitary n-dimensional white noise. This is the so-called diffusive approximation of the rescaled process. This highlights the fact that $N$ is indeed a scale parameter controlling the level of stochasticity of the trajectories and hence their smoothness: when $N$ is large, the individual erratic behaviour tends to be averaged when considering the rescaled abundances.

General diffusive models that are not necessarily limits of sIBM models are widely used to model ecosystems. They are expressed as

|  | $dx=F(x)dt+G(x)dW$ | (2) |
| --- | --- | --- |

They correspond to situations where species form abundant populations that follow a deterministic drift $F(x)$ perturbed by stochastic fluctuations whose variance-covariance matrix is ${G(x)}^{T}G(x)$. The Ricker model considered in this paper is such a model.

Ultimately, as $N$ goes to infinity, the diffusive approximation (1) will converge toward a deterministic ODE model of the form

|  | $\frac{dx}{dt}=f(x)$ | (3) |
| --- | --- | --- |

Deterministic ODE models are also widely used for ecological modelling; they assume that populations are large enough to make stochasticity in the species density or frequency negligible.

This upscaling is illustrated below on the simulation of two SOI models with 10 species. The same parameters are used for both models. The first one has a maximum size N of 100, the second one has a maximum size of $10^{6}$. The trajectories are displayed in Figures B and C for 3000 time steps. As expected, the rescaled abundances for the second case form almost deterministic trajectories.


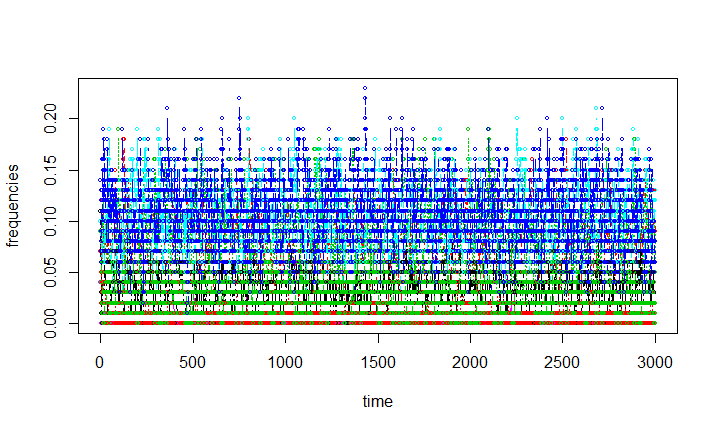


Figure B: SOI simulation with 10 species, 100 individuals and 3000 time steps.


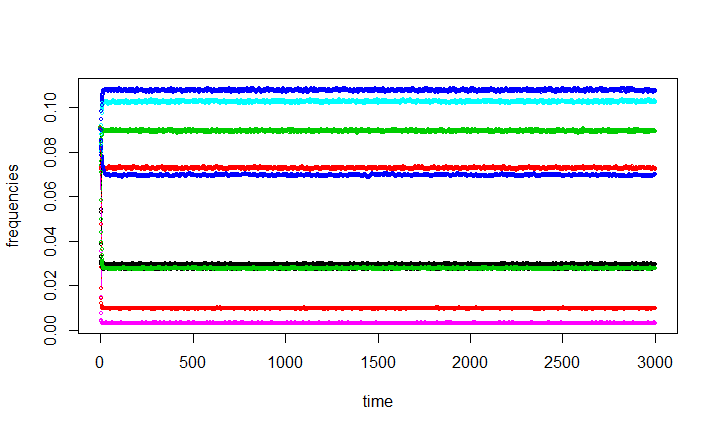


Figure C: SOI simulation with 10 species, 1000000 individuals and 3000 time steps.

The approximation results (1) and (3) are standard results from stochastic process theory that can be found in (Van Kampen 1992), their rigorous mathematical derivation and validity conditions can be found in (Ethier 2009, Darling 2008). They have been applied in many situations in molecular biology and ecology, see e.g. (Lestas 2008, Sloan 2006). The relationships between models are summarized in Figure 1 in the main text.

# Basics of power spectrum analysis

We first recall a few notions on Fourier transform and sampling. First, we are interested in causal signals $x$, that is signals defined on $[0,\infty[$ and equal to zero otherwise. The set of integrable signals, that is signals whose norm is integrable over $\mathbb{R}^{+}$ is denoted $L^{1}(\mathbb{R}^{+})$.

#### 1. Deterministic signals.

The Fourier transform of a deterministic causal signal $x$ in $L^{1}(\mathbb{R}^{+})$ is defined as

$$\hat{x}(\nu)=\hat{x}(s)=\int_{0}^{\infty} e^{-st}x(t)dt$$

with $s=i2\pi\nu$. Note that $\hat{x}(-\nu)=\overline{\hat{x}(\nu)}$, so that we focus on positive frequencies $\nu\geq0$.

The Fourier transform can be extended to more general functions in $L^{2}\left( \mathbb{R}^{+} \right).$

The following proposition relates the flatness of the signal at 0 to the asymptotic decay of the norm of the Fourier transform.

**Proposition 1.** Consider a signal $x$ such that $x$ and its first $n$ derivatives are integrable on $\mathbb{R}^{+}$, with right derivatives at 0 satisfying $x^{'}(0)=\ldots=x^{(n-1)}(0)=0$. Then there exists $\alpha\geq n$ such that the Fourier transform of $x$ satisfies

$$|\hat{x}(\nu)|\in O\left( \frac{1}{\nu^{\alpha}} \right)$$

Consider a deterministic signal $x$ with a well defined Fourier transform, its power spectrum is the square modulus of its Fourier transform

|  | $S(\nu)=\vert\hat{x}(\nu)\vert^{2}$ | (3) |
| --- | --- | --- |

It coincides with the Fourier transform of the autocorrelation function

|  | $R(\tau)=\int_{0}^{\infty} x(t)\bar{x}(t-\tau)dt.$ | (4) |
| --- | --- | --- |

#### 2. Stochastic signals.

For a stochastic signal $x$ generated by a weakly stationary, ergodic process, the autocorrelation is defined as

|  | $R(\tau)=\mathbb{E\{}x(t)\bar{x}(t-\tau)\}=\lim_{T\to\infty} \frac{1}{T}\int_{0}^{T} x(t)\bar{x}(t-\tau)dt$ | (5) |
| --- | --- | --- |

The Fourier transform of $R$, provided it exists, is by definition the power spectrum of $x$.

|  | $S(\nu)=\hat{R}(\nu)$ | (6) |
| --- | --- | --- |

If $x$ itself has a Fourier transform in the stochastic sense, that is the mean square integral $\hat{x}_{T}(\nu)=\frac{1}{\sqrt{T}}\int_{0}^{T} x(t)e^{-i2\pi\nu t}dt$ is well defined, then the following generalization of (3) holds:

|  | $S(\nu)=\lim_{T\to\infty} \mathbb{E}\{\vert\hat{x}_{T}(\nu)\vert^{2}\}$ | (7) |
| --- | --- | --- |

#### 3. Computing and interpreting the slope of the log-power spectrum.

As a consequence of proposition 1, the asymptotic slope of the log/log plot of the power spectrum of a signal is related to the smoothness and the “flatness” (the number of null derivatives) of the auto-correlation function as $\tau\to0$. The longer the auto-correlation, the flatter the auto-correlation function at zero and the steeper the slope of the power spectrum.

For stochastic signals, the auto-correlation of a pure white noise is the Dirac measure, its spectrum is equal to a constant value on all frequencies, so the asymptotic slope of the log/log plot is zero. The power spectrum of a trajectory in a Wiener process (Brownian motion), which is the integral of a white noise, is shown to have a log/log slope of -2 (brown noise).

For linear time invariant SDE with a stable drift matrix and deterministic initial condition, there is an explicit formula for the power spectrum. It can be decomposed into two contributions, a deterministic part coming from the initial condition and a stochastic part coming from the intrinsic noise. So according to the noise level, the deterministic or the stochastic part will dominate the available frequency range, resulting in slopes less than -2 when the deterministic part dominates and closer to -2 as the noise level increases. There is no explicit formula for nonlinear SDE systems, but by analogy it is clear that a higher noise level will induce the same shifts on the slopes.

The log/log slope of the power spectrum for signals generated by Markov jump processes lie between 0 and -2 (pink noise). The power spectrum of signals originating from stable linear time invariant (LTI) ODE systems have a log/log slope less than -2 (black “noise”). This also holds for general stable ODE systems (3).

#### 4. Limits related to practical implementation.

In practice, the observed signals are sampled with period $p$ and observed on a finite horizon $T$. The power spectrum is then computed as the discrete Fourier transform of the estimated auto-correlation of the truncated signal. Prior to spectrum estimation, the signal is usually “detrended”, meaning that the linear trend is removed, in order to avoid large peaks at zero frequency.

The truncation of a signal tends to mix the contributions of different frequencies, causing what is known as "spectral leakage". Spectral analysis tools use windowing techniques to mitigate this phenomenon, however shorter time horizons will degrade the power spectrum reconstruction.

Another critical issue in practical implementation may arise from sampling. Indeed, the approximated power spectrum may be distorted by aliasing if the sampling frequency is less than half the bandwidth of the true unsampled signal. This would result in an apparent shift of the asymptotic slope of the power spectrum towards zero.

# 5. Power spectrum slope computation.

All the notions that were introduced above are relevant for the *asymptotic* slope of the power spectrum, except when dealing with constant or pure white or brown noise signals, for which the power spectrum is flat or proportional to $\frac{1}{f^{2}}$ for all frequencies.

However, many signals, among which deterministic signals produced by linear differential equations with constant coefficients, are known to experience slope changes in their spectrum. Moreover, the power spectrum is corrupted by aliasing and numerical noise at high frequencies.

As a consequence, the power spectrum slope cannot be estimated by a simple linear regression on the power spectrum. To overcome this, we implemented a spline smoothing on the spectrum (log10 of power as a function of log10 of frequencies), using the smooth.spline function in R and estimated the slope as the minimal value of the derivative of the spline. The amount of smoothing is controlled by a parameter df, such that low df values correspond to high smoothing and conversely high df values lead to weak smoothing. After numerical inspection, we found that $df=max(2, log10\left( length(time series) \right)$ nicely adjusts the amount of smoothing when the length of the series varies.

# 6. Comparison of model-specific time scales.

To check whether our default sampling period of one is appropriate when comparing the power spectrum slopes of time series generated with different models, we computed the upper bound of the sampling period, i.e. the largest sampling period that does not lead to under-sampling. This was done on the noise free versions of the gLV and Ricker models. The models were linearized around the mean asymptotic value and we used a standard analysis of the systems time scales based on the eigenvalues of the Jacobian matrix in the linearized model (see formula in the caption of Figure D) to determine the upper bound of the sampling period that would prevent aliasing. Since this upper bound is larger than one in all but one of the tested time series, a bias due to under-sampling can be excluded.

For fully stochastic models like Hubbell and SOI it is not easy to disentangle time scale effects and intrinsic stochastic noise. Considering only the demographic (growth and extinction) effects, we analysed the deterministic large population limit as above, and the upper bound of the corresponding sampling period is displayed in Figure D. However, in the Hubbell and SOI models, a stochastic immigration occurs, acting like an external input that influences the system dynamics. If the immigration events are important but not time correlated (which is the case in Hubbell and SOI), and the sampling period misses many of them, the samples will appear more uncorrelated and the noise colour will shift accordingly. This is illustrated by the noise colour percentage in series 14, 46 and 49 corresponding to the same generating model (Hubbell, same parameters) sampled with a period of 1, 10 and 5 days, respectively. We observe a clear shift from brown to pink in the noise colour, corresponding to an increasing amount of uncorrelated immigration events (with a period of one day) missed when under-sampling.

**Figure D**: The upper bounds of the sampling periods are plotted for selected time series. For gLV and Ricker, an approximated upper bound for the sampling period in the noise-free models was computed as follows: For gLV, we considered the linearized model around the asymptotic steady state or around the asymptotic mean state in case of oscillations. The upper bound is computed as

$$T_{upper}=\pi\left( \max_{i} \left( \text{max}\left( \left| \text{real}\left( \lambda_{i} \right) \right|,\left| \text{imag}\left( \lambda_{i} \right) \right| \right) \right) \right)^{-1}$$

where the $\lambda_{i}$ ‘s are the eigenvalues of the Jacobian matrix in the linearized model. For sampling periods above this upper bound, some components in the deterministic signal could be altered. For the Ricker model, which is a discrete time model, we considered the linearized discrete time model and applied the same formula to the logarithm of the eigenvalues of the Jacobian matrix. For the Hubbell and SOI models, which are stochastic models, we considered the deterministic limit of the birth-death parts only and performed the same computation as above, so that the computed bound does not account for noise induced by stochastic immigration and interactions. For visualization, values above 10 are set to 10 and day one is indicated with a dashed line.

**References**

Solé, R. V., & Bascompte, J. (2006). Self-Organization in Complex Ecosystems.(MPB-42) (Vol. 42). Princeton University Press.

Fisher, C. K., & Mehta, P. (2014). Identifying keystone species in the human gut microbiome from metagenomic timeseries using sparse linear regression. PloS one, 9(7), e102451.

Washburne A. D., Burby J. W., & Lacker D. (2016). Novel Covariance-Based Neutrality Test of Time-Series Data Reveals Asymmetries in Ecological and Economic Systems. *PLoS Computational Biology* **12:** e1005124.

Hui, C. (2006). Carrying capacity, population equilibrium, and environment's maximal load. *Ecological Modelling*, *192*(1), 317-320.

Solé, R. V., Alonso, D., & McKane, A. (2002). Self–organized instability in complex ecosystems. Philosophical Transactions of the Royal Society of London B: Biological Sciences, 357(1421), 667-681.

Van Kampen, N. G. (1992). Stochastic processes in physics and chemistry (Vol. 1). Elsevier.

Ethier, S. N., & Kurtz, T. G. (2009). Markov processes: characterization and convergence (Vol. 282). John Wiley & Sons.

Darling, R. W. R., and Norris, J. R. (2008). Differential equation approximations for Markov chains. Probability surveys, 5, 37-79.

Lestas, I., Paulsson, J., Ross, N. E., & Vinnicombe, G. (2008). Noise in gene regulatory networks. IEEE Transactions on Automatic Control, 53(Special Issue), 189-200.

Sloan, W. T., Lunn, M., Woodcock, S., Head, I. M., Nee, S., & Curtis, T. P. (2006). Quantifying the roles of immigration and chance in shaping prokaryote community structure. Environmental microbiology, 8(4), 732-740.
