# Supplementary figures and images for: Signatures of ecological processes in microbial community time series
Source: Microbiome. 2018 Jun 28;6:120. doi: 10.1186/s40168-018-0496-2 (PMC6022718; doi:10.1186/s40168-018-0496-2)

a) Maximum auto-correlation

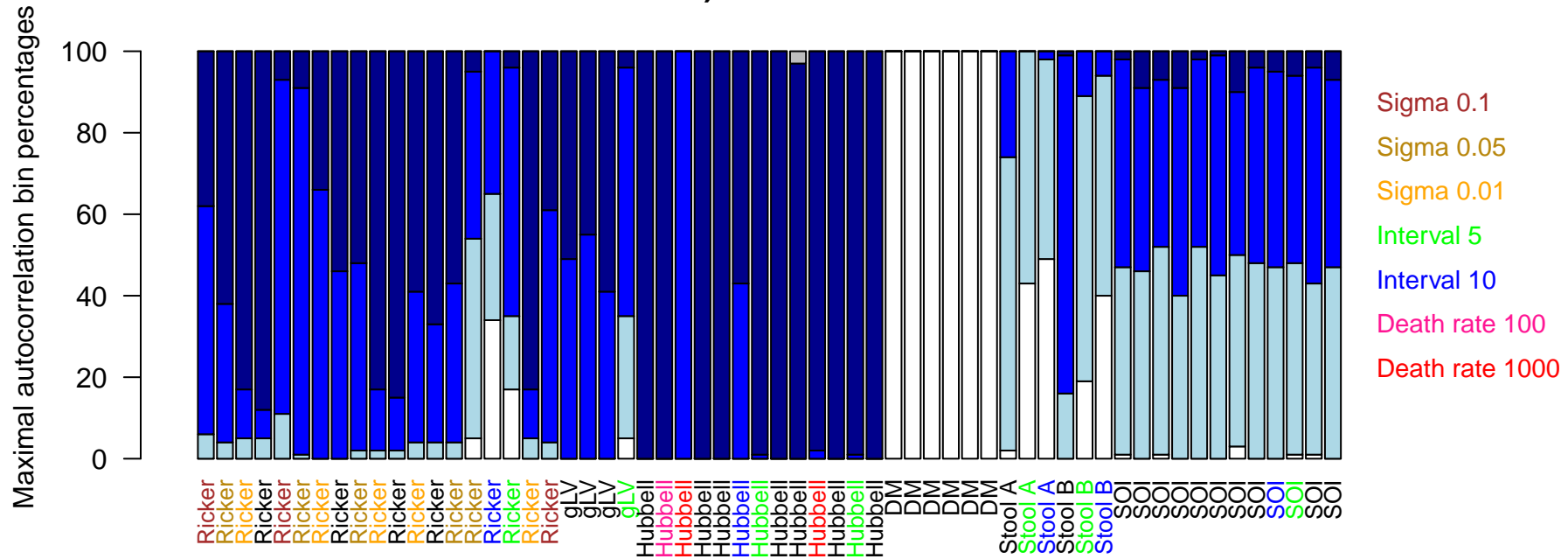

b) Hurst exponent

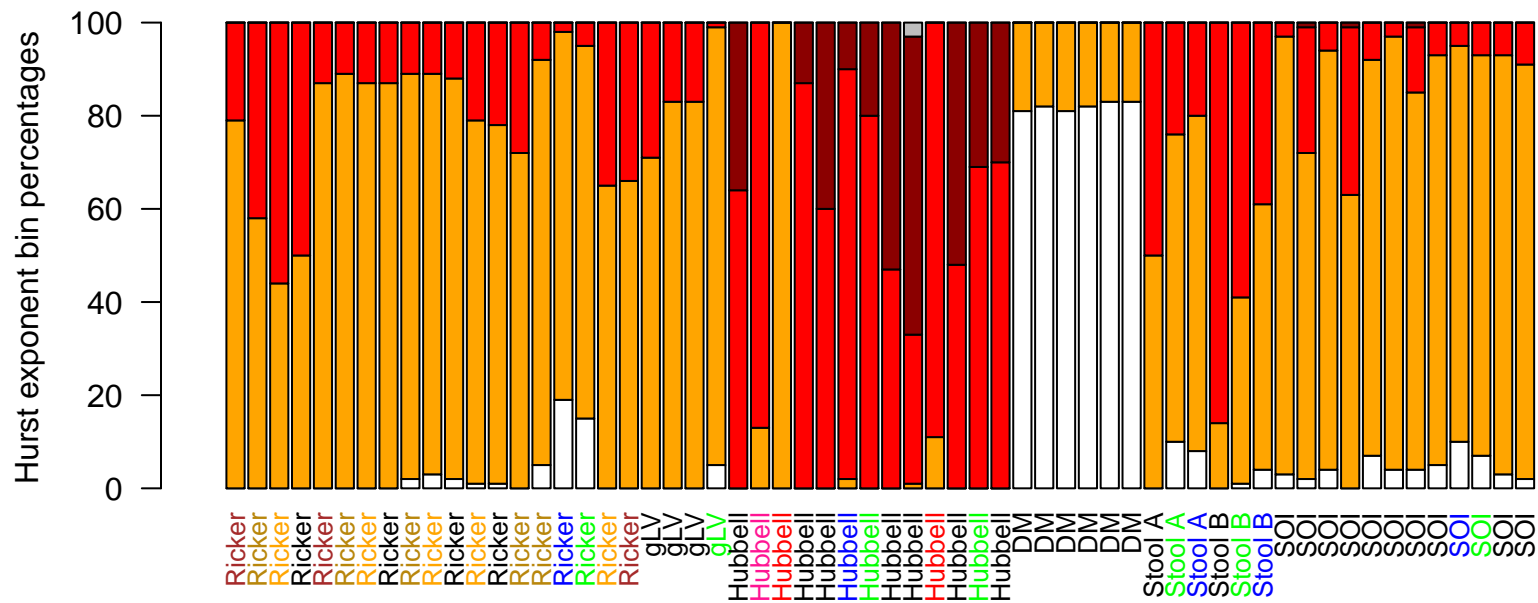

Supplement: Supplementary file 3 — Figure S1. Maximal autocorrelation and Hurst exponent profiles reproduce patterns seen with noise types. (a) The species in each time series are grouped in four bins according to their maximum (lagged) autocorrelation (white: below 0.3, light blue: 0.3 to 0.6, blue: 0.6 to 0.95, dark blue: above 0.95). (b) The species are separated into four Hurst exponent bins, ranging from white (below 0.6), orange (0.6 to 0.8), red (0.8 to 0.9) to dark red (above 0.9). Species for which the maximum autocorrelation or Hurst exponent could not be computed (due to a large number of zeros) are colored in gray. Labels for time series are colored according to the level of non-zero intrinsic noise (sigma) for Ricker, according to the death rate if larger than one for Hubbell, according to the interval if larger than one (with interval coloring taking precedence over sigma) and black otherwise. (PDF 9 kb) [file 40168_2018_496_MOESM3_ESM.pdf]

**a) Ricker model, PEP=20, sigma=0.05**

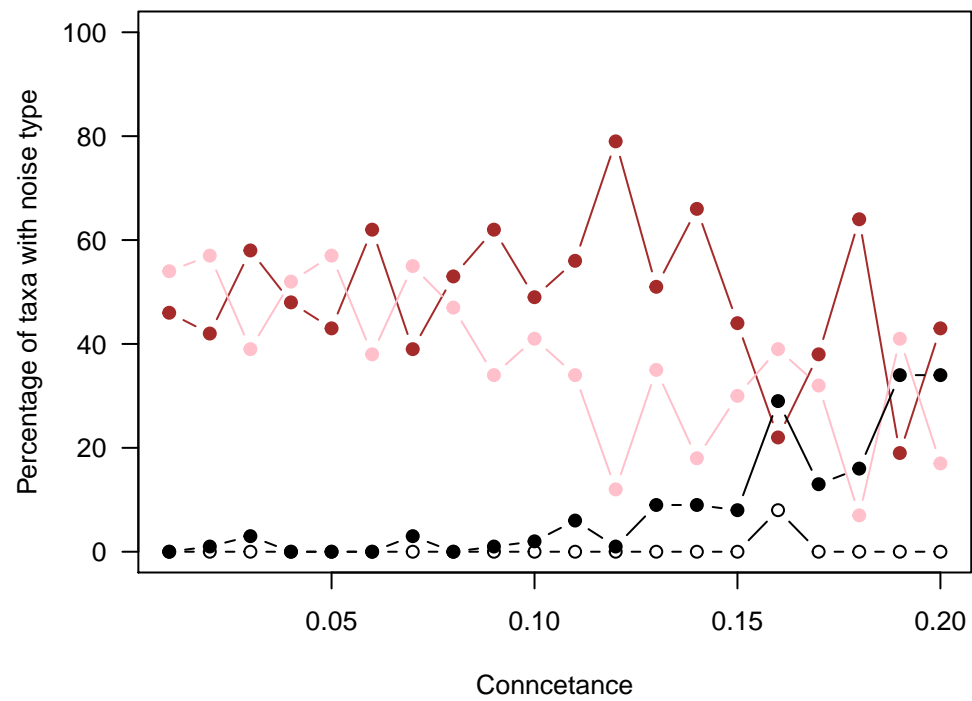

**b) Ricker model, c=0.01, sigma=0.05**

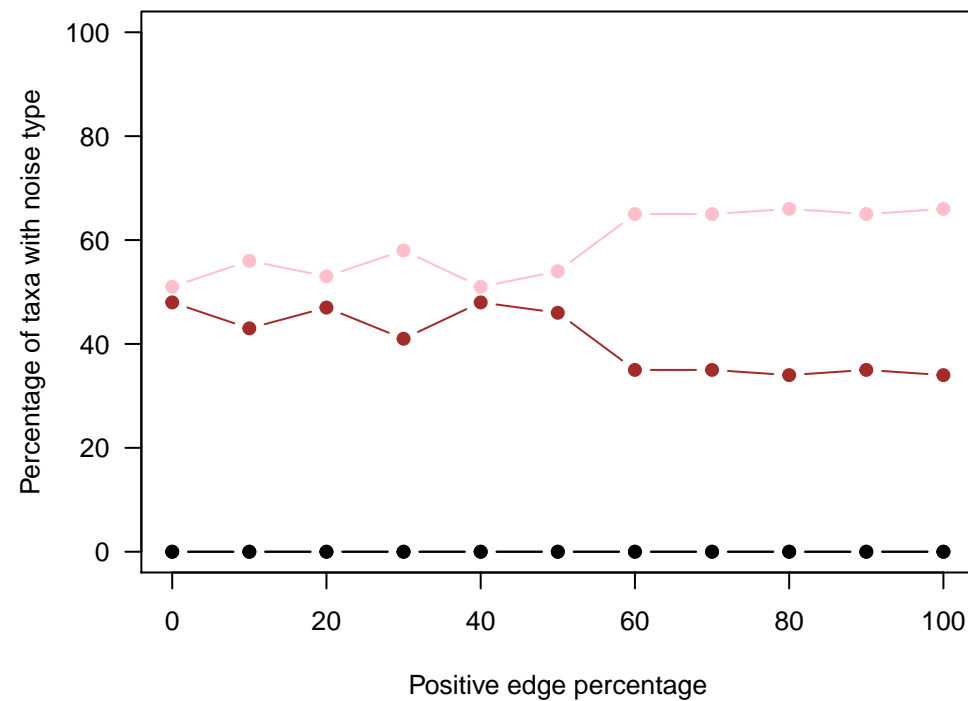

**c) gLV model, PEP=20**

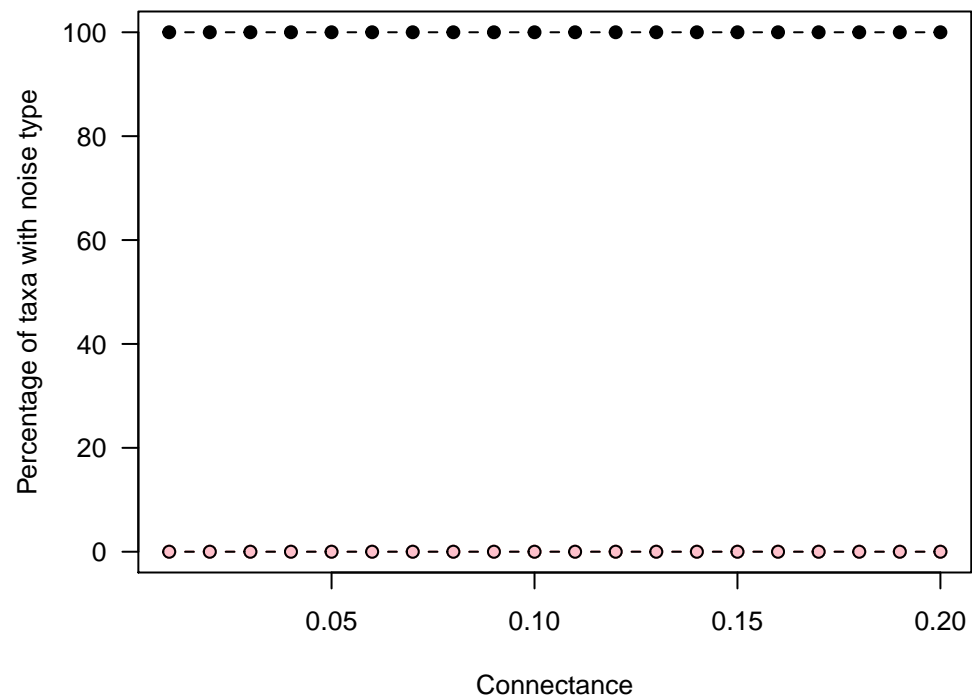

**d) gLV model, c=0.01**

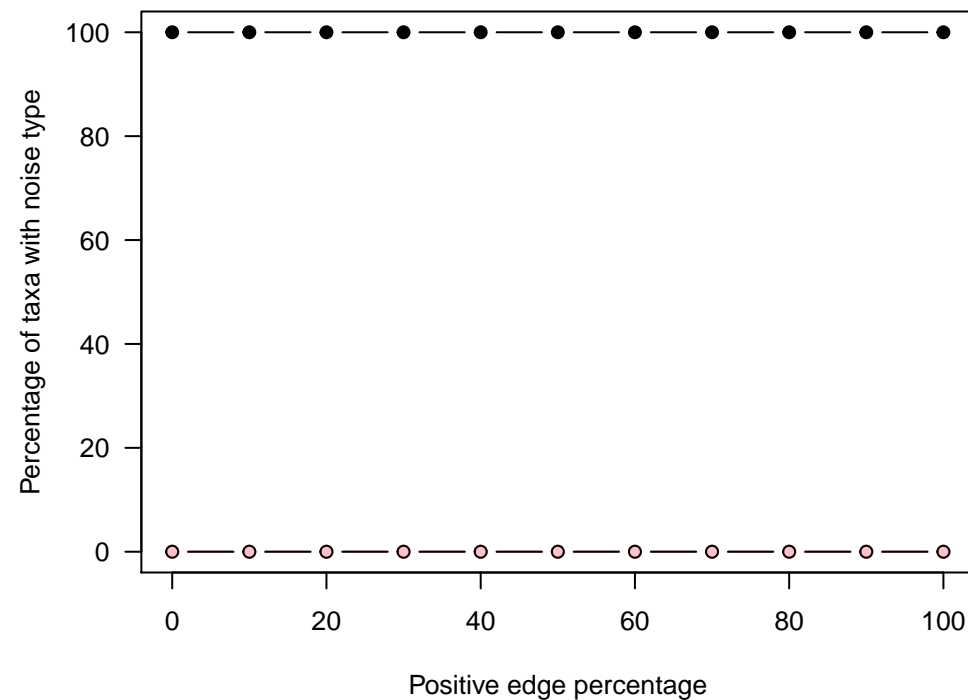

Supplement: Supplementary file 4 — Figure S2. The noise-type classification and the neutrality test for Ricker and gLV are robust to positive edge percentage, but connectance affects noise types in Ricker. (a, c) The percentage of taxa with black, brown, pink and white noise types is plotted against the connectance of the interaction matrix for Ricker and gLV, respectively. The percentage of black taxa in Ricker was positively correlated to connectance (Spearman’s rho: 0.86, p value < 0.00001), whereas the percentage of pink taxa in Ricker was negatively correlated to connectance (Spearman’s rho: − 0.71, p value = 0.00049). (b, d) The percentage of taxa with black, brown, pink, and white noise types is plotted against the positive edge percentage of the interaction matrix for Ricker and gLV, respectively. All neutrality test p values were zero, indicating non-neutral dynamics. Time series were generated for 100 species and 3000 time points. (PDF 16 kb) [file 40168_2018_496_MOESM4_ESM.pdf]

**a) SOI model, PEP=20**

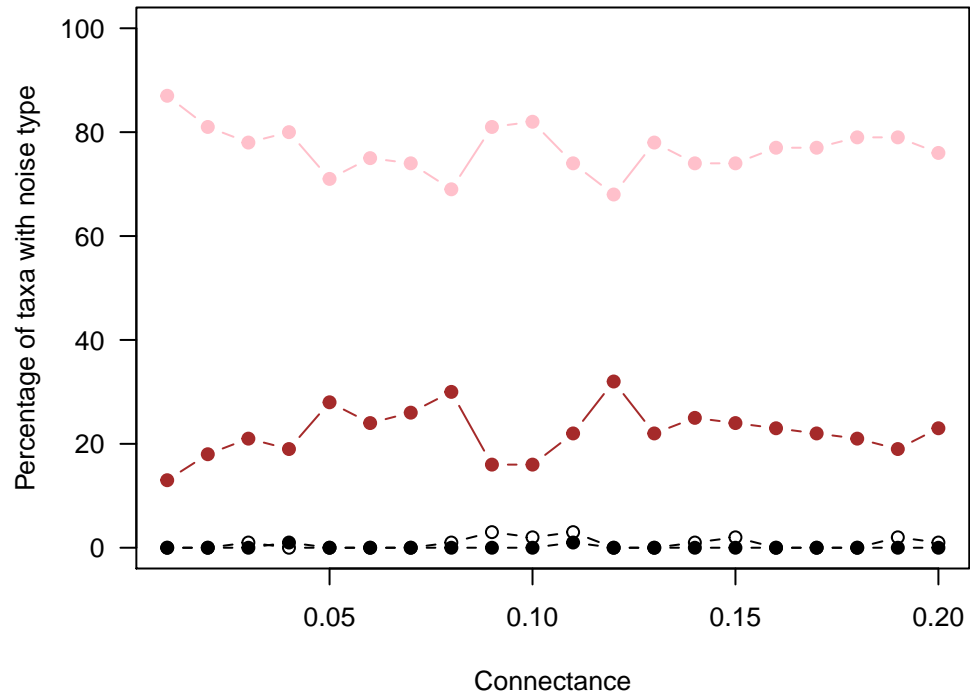

**b) SOI model, c=0.01**

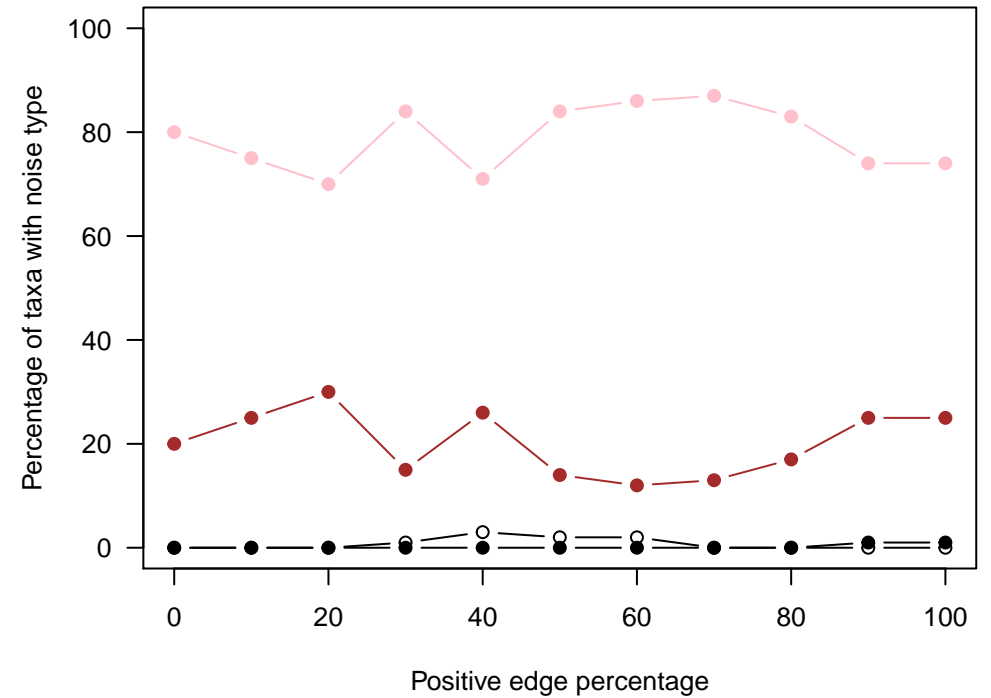

**c) SOI model, PEP=20**

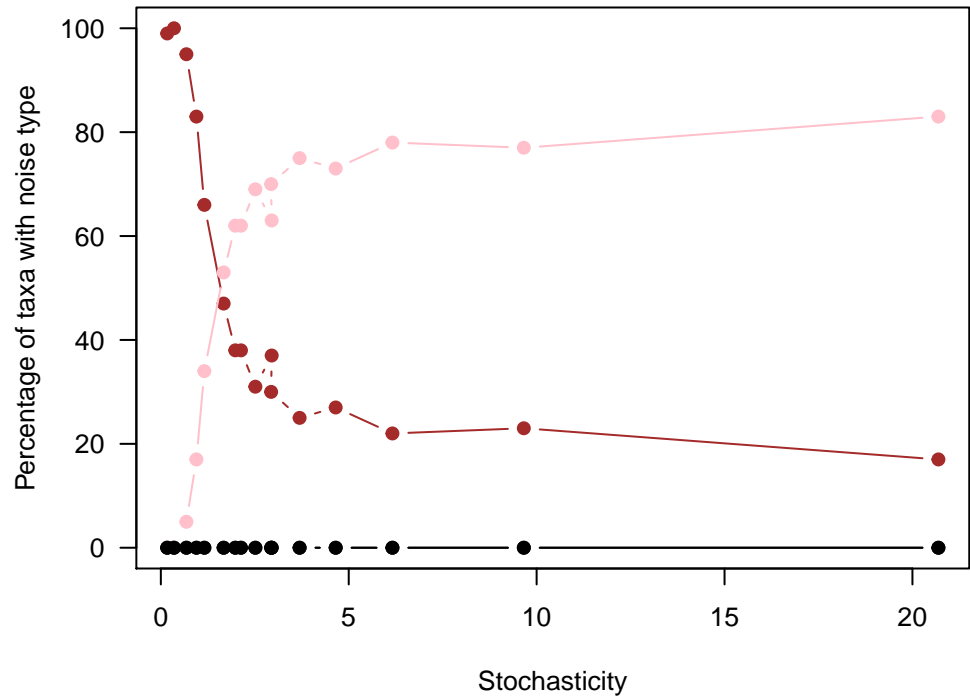

**d) SOI model, PEP=20**

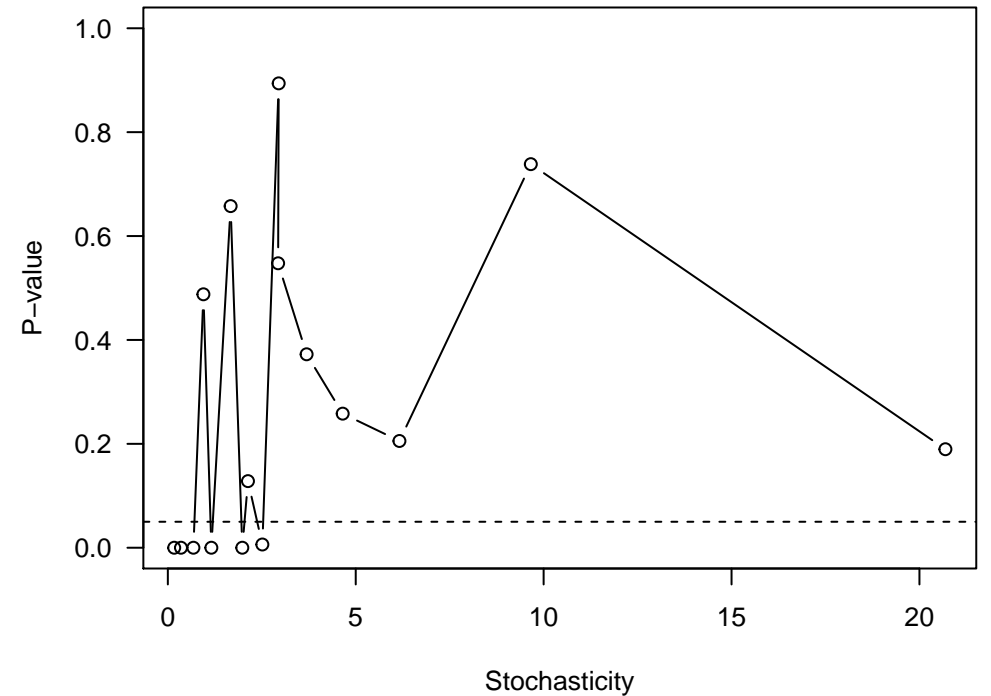

Supplement: Supplementary file 5 — Figure S3. The noise-type classification and the neutrality test for SOI are robust to interaction matrix properties. (a) The percentage of taxa with black, brown, pink, and white noise types is plotted against the connectance of the interaction matrix. (b) The percentage of taxa with black, brown, pink and white noise types is plotted against the positive edge percentage of the interaction matrix. The stochasticity in the SOI model is plotted against (c) noise types and (d) neutrality p values. Stochasticity is defined here as the ratio between the mean of extinctions and immigrations and the mean of the absolute interaction strengths (excluding diagonal values). Neutrality test p values for (a) and (b) were zero, indicating non-neutral dynamics. Time series were generated for 100 species and 3000 time points. (PDF 16 kb) [file 40168_2018_496_MOESM5_ESM.pdf]

a) Noise types absolute abundances

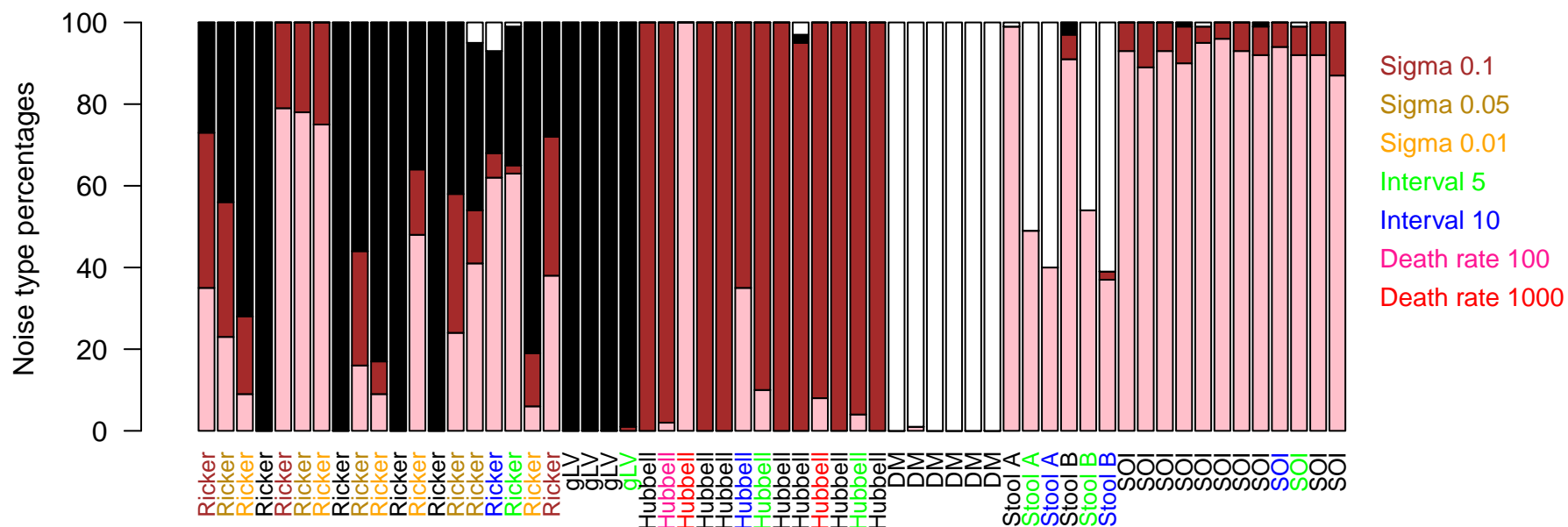

b) Noise types last 100 time points

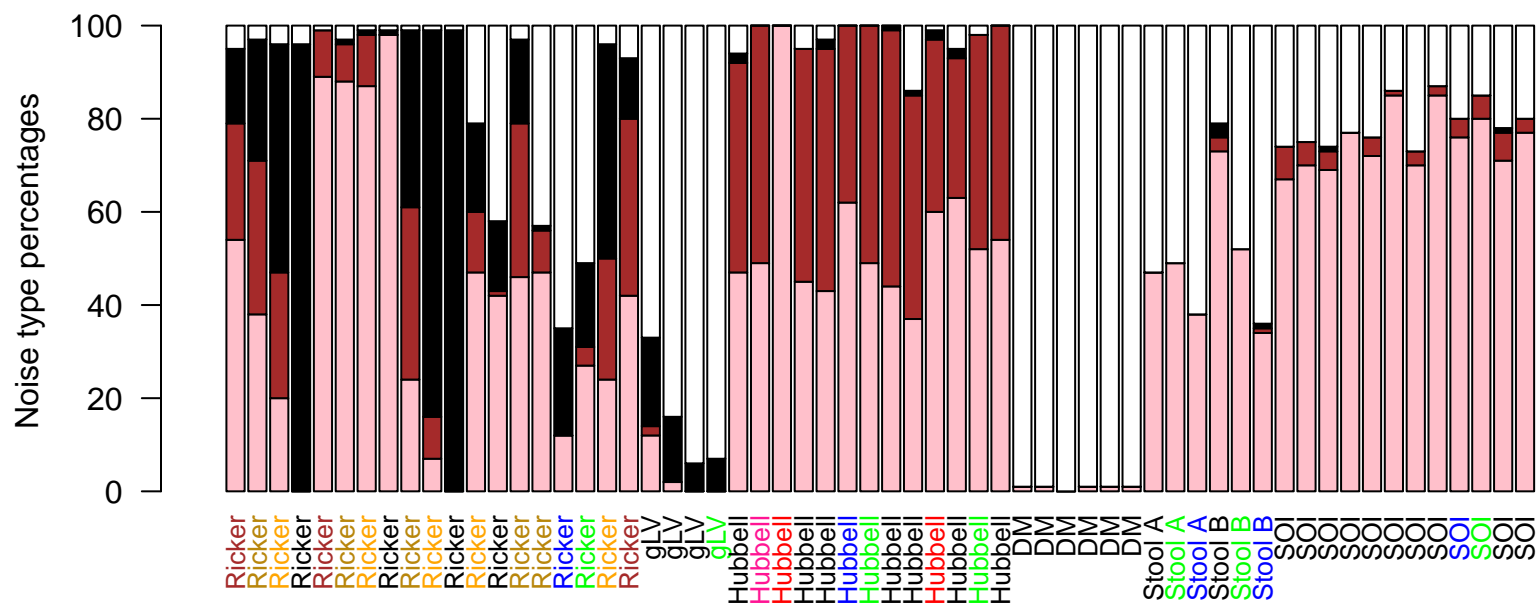

Supplement: Supplementary file 7 — Figure S5. The test for temporal structure with noise types is robust to compositionality and the absence of transient dynamics. (a) The noise-type profiles for absolute abundances do not differ noticeably from those for relative abundances shown in Figure 3a. (b) When noise types are computed for the last hundred time points, most time series are correctly classified as temporally structured or unstructured. Labels for time series are colored according to the level of non-zero intrinsic noise (sigma) for Ricker, according to the death rate if larger than one for Hubbell, according to the interval if larger than one (with interval coloring taking precedence over sigma) and black otherwise. (PDF 8 kb) [file 40168_2018_496_MOESM7_ESM.pdf]

a) Noise types Poisson

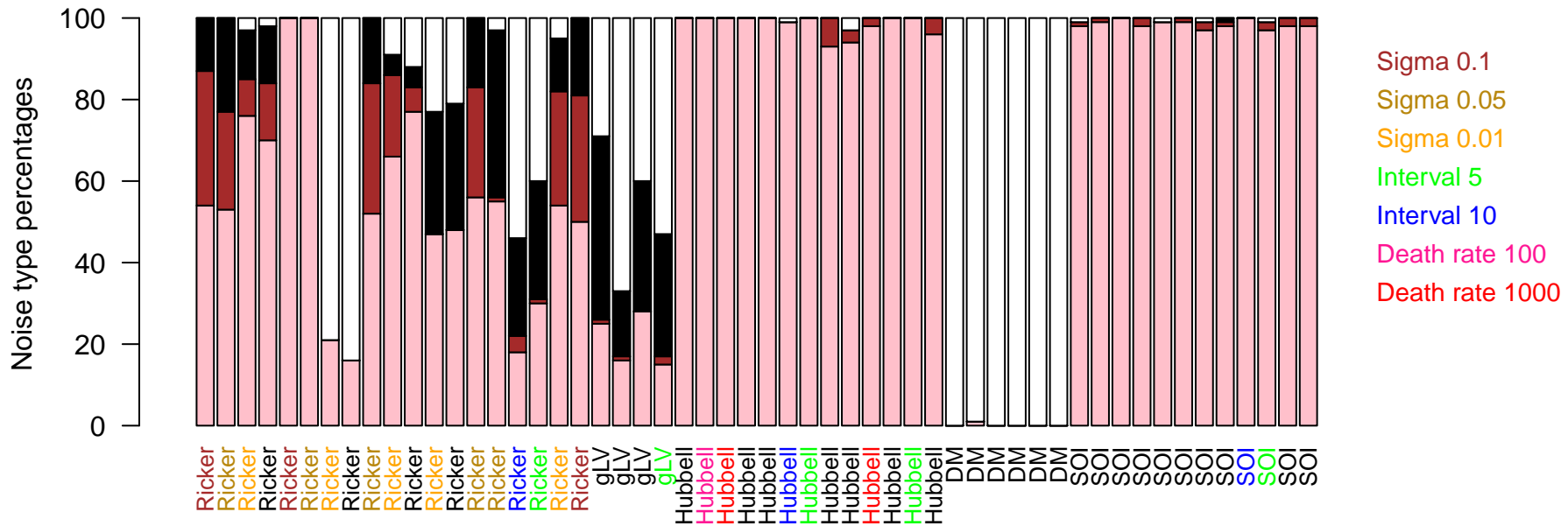

b) Noise types Multinomial

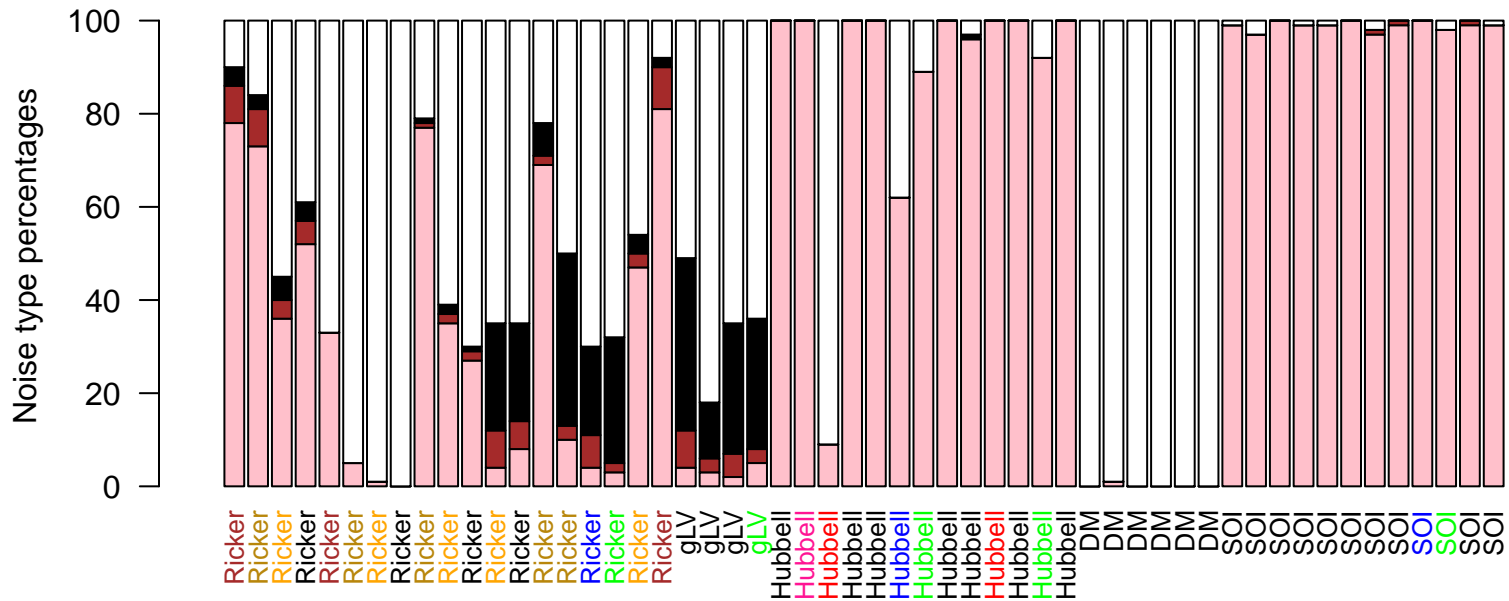

Supplement: Supplementary file 8 — Figure S6. The test for temporal structure with noise types is robust to noise. (a) Noise-type profile in the presence of noise generated with the Poisson distribution for each species and each sample. (b) Noise-type distribution in the presence of noise generated with the multinomial distribution for each sample. Labels for time series are colored according to the level of non-zero intrinsic noise (sigma) for Ricker, according to the death rate if larger than one for Hubbell, according to the interval if larger than one (with interval coloring taking precedence over sigma) and black otherwise. (PDF 8 kb) [file 40168_2018_496_MOESM8_ESM.pdf]

### a) Noise types (10 time points)

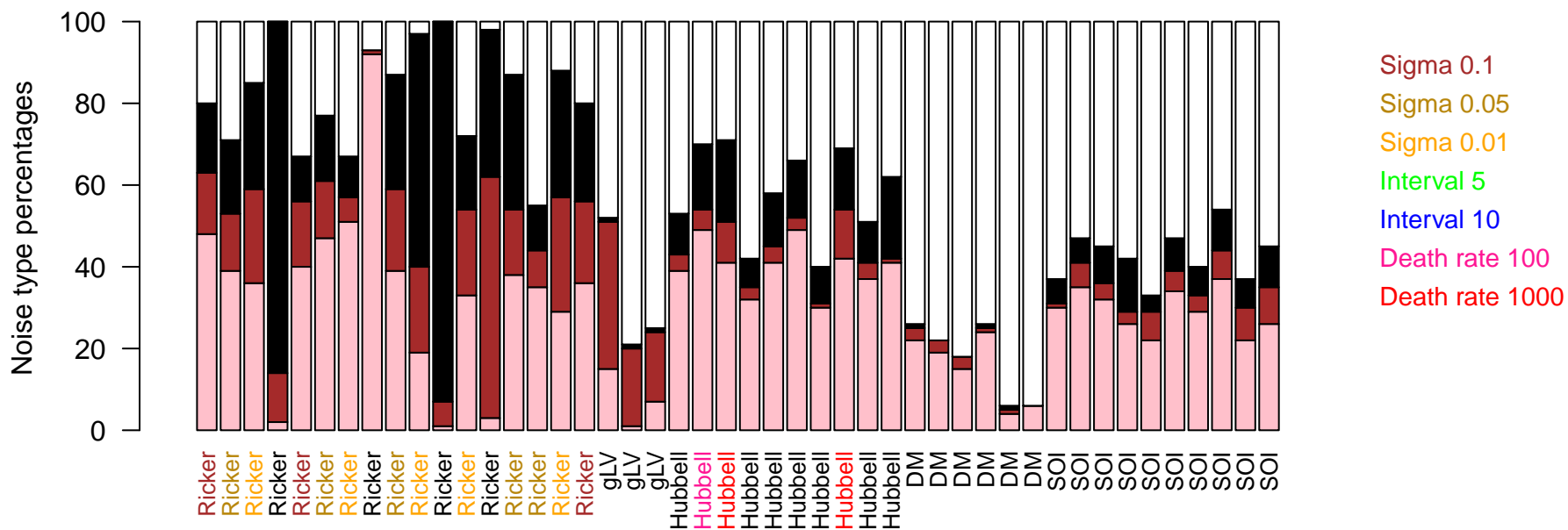

### b) Noise types (25 time points)

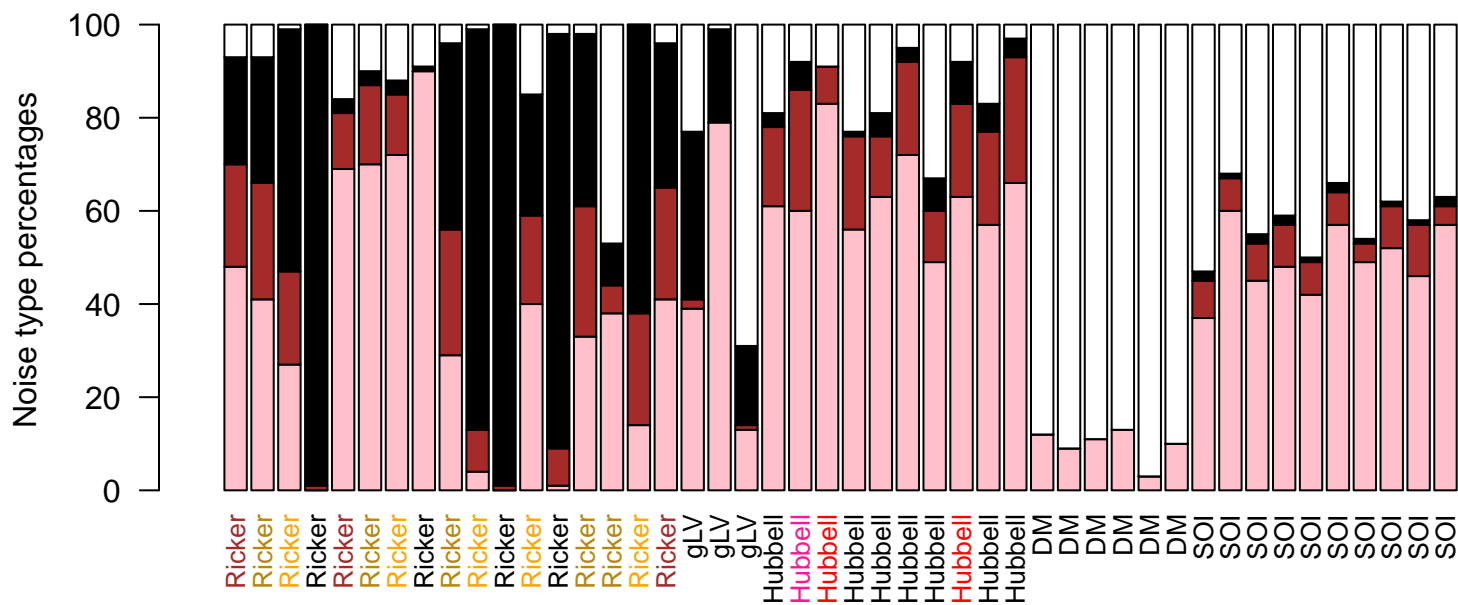

Supplement: Supplementary file 9 — Figure S7. Increasing the time series length improves the accuracy of the test for temporal structure. Noise types were computed for time series sub-sets from 1000 to 1010 (a) and 1000 to 1025 (b) for all data sets with more than 1000 time points. Labels for time series are colored according to the level of non-zero intrinsic noise (sigma) for Ricker, according to the death rate if larger than one for Hubbell, according to the interval if larger than one (with interval coloring taking precedence over sigma) and black otherwise. (PDF 8 kb) [file 40168_2018_496_MOESM9_ESM.pdf]

a) Noise types (50 time points)

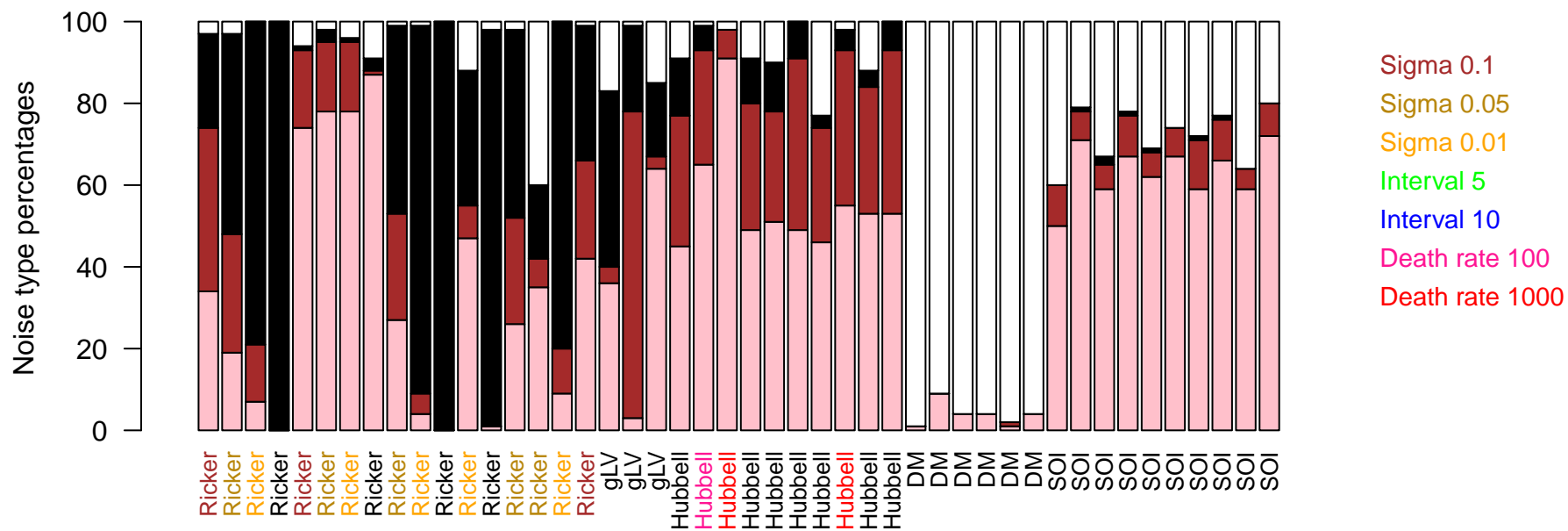

b) Noise types (100 time points)

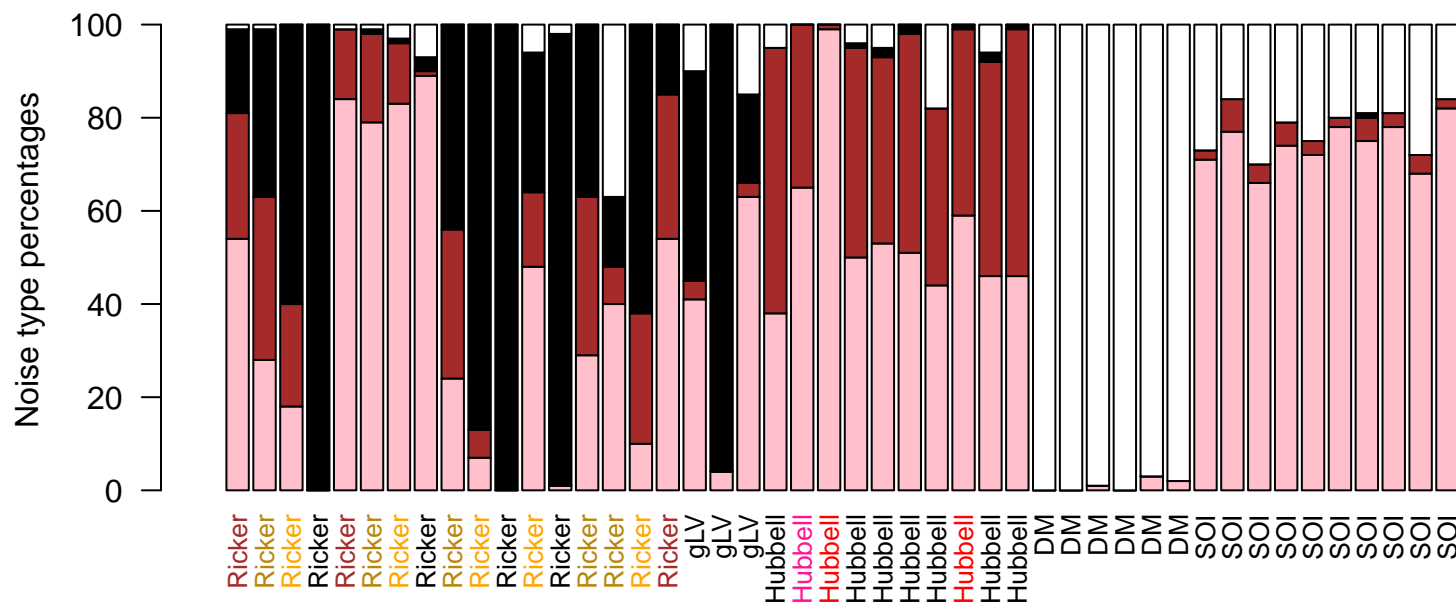

Supplement: Supplementary file 10 — Figure S8. Increasing the time series length improves the accuracy of the test for temporal structure. Noise types were computed for time series sub-sets from 1000 to 1050 (a) and 1000 to 1100 (b) for all data sets with more than 1000 time points. Labels for time series are colored according to the level of non-zero intrinsic noise (sigma) for Ricker, according to the death rate if larger than one for Hubbell, according to the interval if larger than one (with interval coloring taking precedence over sigma) and black otherwise. (PDF 7 kb) [file 40168_2018_496_MOESM10_ESM.pdf]

**Community time series Stool A**

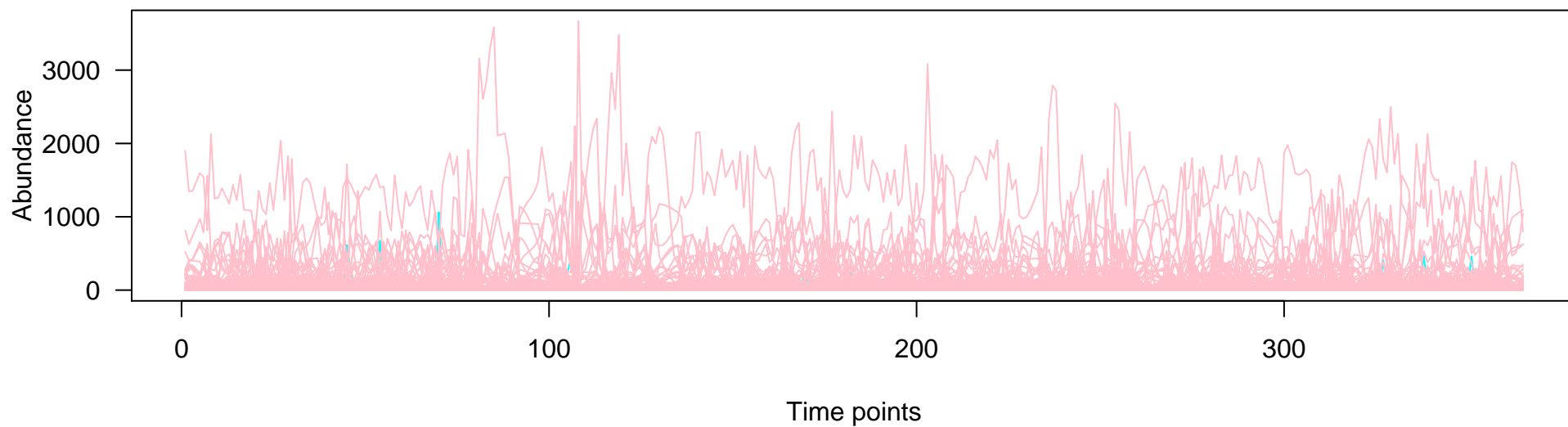

**Community time series Stool B**

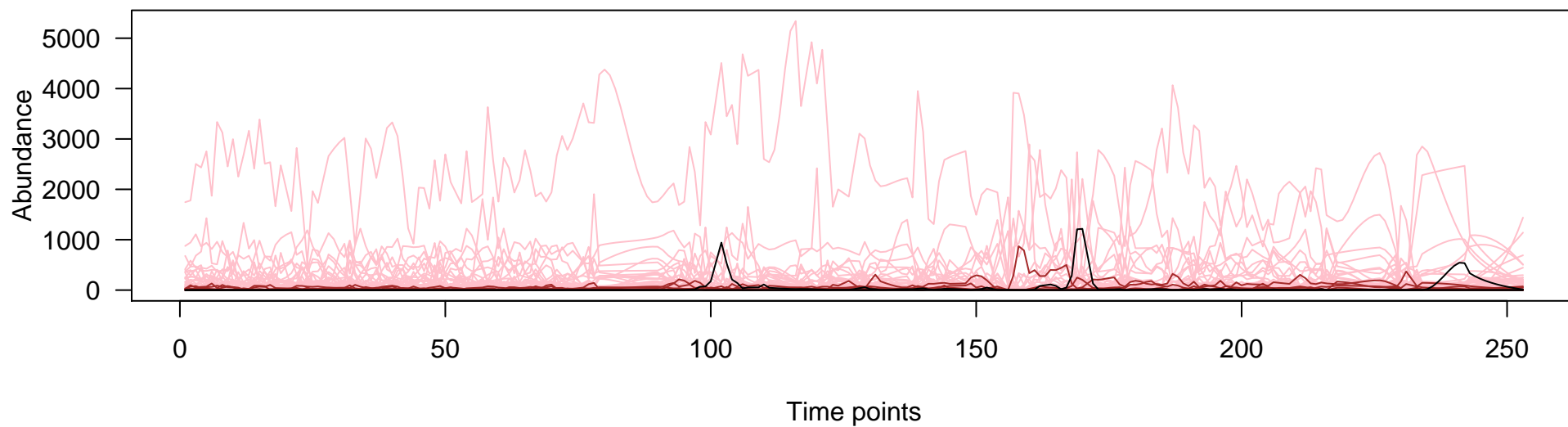

Supplement: Supplementary file 13 — Figure S11. Time series of the 100 top abundant OTUs in the processed stool data of individual A and B [3]. The OTUs are colored according to their noise type (with cyan for white noise). (PDF 17 kb) (PDF 219 kb) [file 40168_2018_496_MOESM13_ESM.pdf]

Noise type classification for stool A across rarefactions

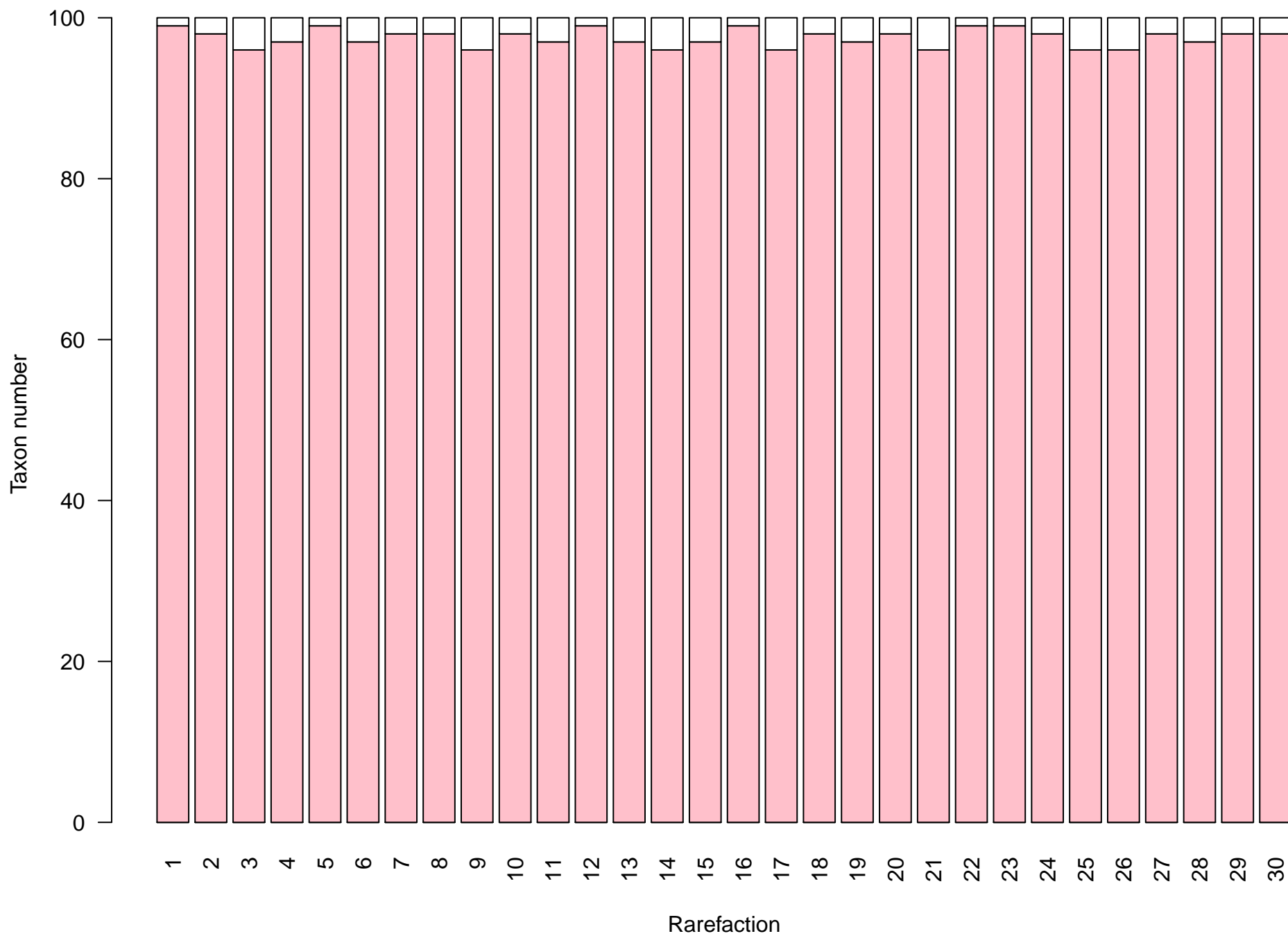

Supplement: Supplementary file 14 — Figure S12. Variability of noise-type classification across rarefactions. The noise types of 100 taxa selected to be top abundant in one rarefaction were computed for repeated rarefactions in the stool data set of individual A [3]. (PDF 5 kb) [file 40168_2018_496_MOESM14_ESM.pdf]
