# Supplementary material for: Signatures of ecological processes in microbial community time series
Source: Microbiome. 2018 Jun 28;6:120. doi: 10.1186/s40168-018-0496-2 (PMC6022718; doi:10.1186/s40168-018-0496-2)
Supplement: Supplementary file 6 — Figure S4. The noise-type classification and the neutrality test are robust for a wide parameter range in the Hubbell model, but noise types are affected by the death rate. (a) The percentage of taxa with black, brown, pink and white noise types is plotted against the death rate. There is a significant negative correlation between the percentage of brown species and the death rate (Spearman’s rho: − 0.85, p value < 0.000001) and a corresponding positive correlation of the percentage of pink species to the death rate (Spearman’s rho: 0.94, p value < 0.000001). (b) The p values of the neutrality test are plotted against the death rate. (c) The percentage of taxa with black, brown, pink, and white noise types is plotted against the number of individuals. (d) The p values of the neutrality test are plotted against the number of individuals. (d) The percentage of taxa with black, brown, pink, and white noise types is plotted against the immigration rate. (e) The p values of the neutrality test are plotted against the immigration rate. Neutrality is rejected for a p value below 0.05. The p value of 0.05 is indicated by a dashed horizontal line. Time series were generated for 100 species and 3000 time points. For the immigration rate, the percentage of noise types of taxa with non-zero abundances was plotted, since for the low immigration rates tested in this simulation, many taxa have abundances of zero. (PDF 40 kb) [file 40168_2018_496_MOESM6_ESM.pdf]

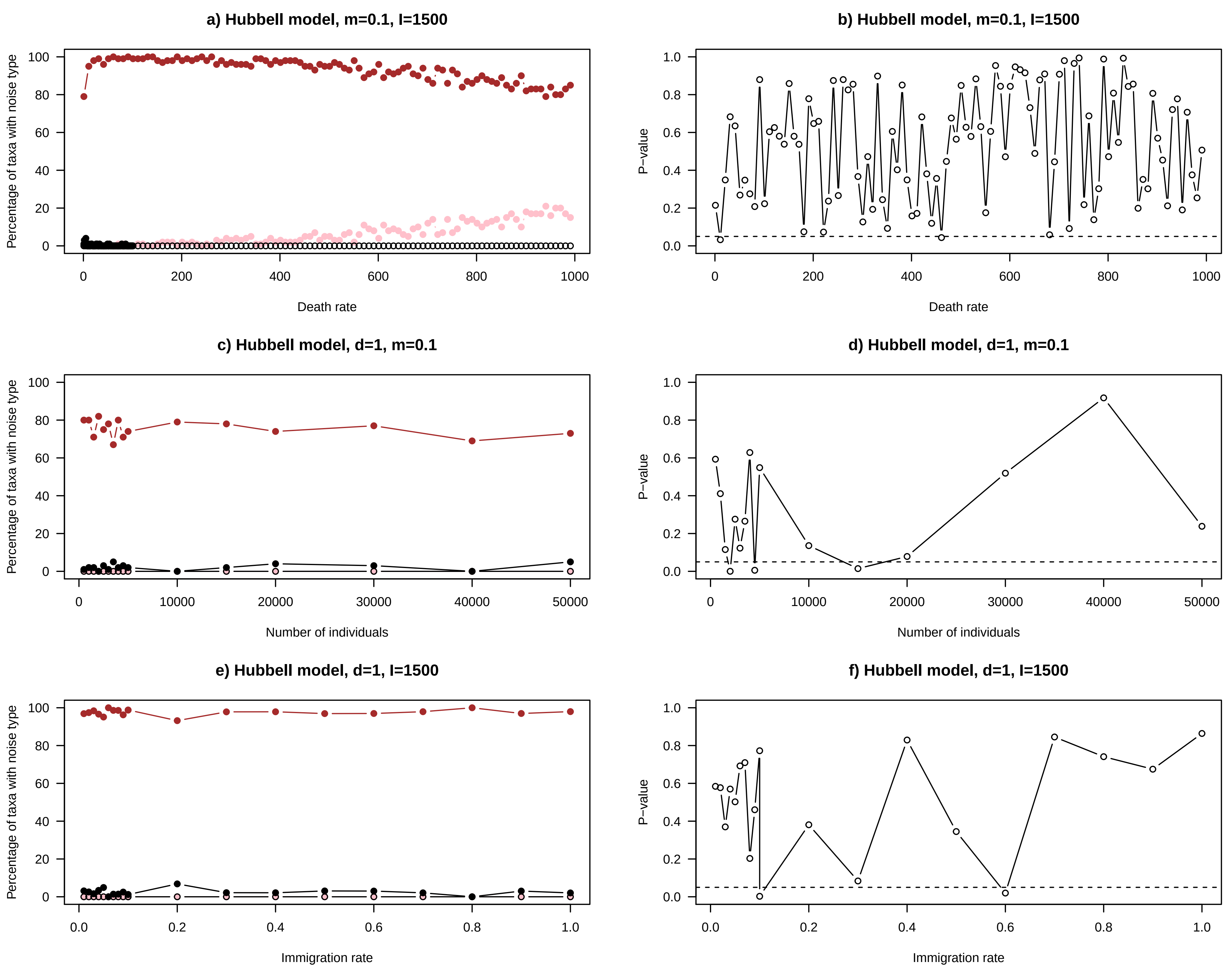

a) Hubbell model, m=0.1, I=1500
Percentage of taxa with noise type
Death rate
b) Hubbell model, m=0.1, I=1500
P−value
Death rate
c) Hubbell model, d=1, m=0.1
Percentage of taxa with noise type
Number of individuals
d) Hubbell model, d=1, m=0.1
P−value
Number of individuals
e) Hubbell model, d=1, I=1500
Percentage of taxa with noise type
Immigration rate
f) Hubbell model, d=1, I=1500
P−value
Immigration rate
