# Supplementary material for: Signatures of ecological processes in microbial community time series
Source: Microbiome. 2018 Jun 28;6:120. doi: 10.1186/s40168-018-0496-2 (PMC6022718; doi:10.1186/s40168-018-0496-2)
Supplement: Supplementary file 11 — Figure S9. The presence of noise decreases the accuracy of the neutrality test but affects network inference accuracy less. (a) For the last 100 time points, when many simulated time series reach equilibrium, neutrality is erroneously rejected for several Hubbell time series and erroneously detected for a number of Ricker and SOI time series. The classification does not change for the stool time series. (b) The addition of Poisson noise does not introduce false negatives in the neutrality test, but introduces false positives (i.e., Hubbell time series for which neutrality is rejected). The dashed lines in (a) and (b) indicate the value corresponding to a p value of 0.05. For values above, neutrality is rejected. (c) LIMITS accuracy, i.e., mean correlation of inferred and known interaction matrix, for time series with Poisson noise. Inference failed for gLV time series. (d) LIMITS goodness of fit for time series with Poisson noise. The goodness of fit was computed as the mean correlation between original and predicted time series. The data points are colored according to the interval in panels (a), (b) and (d), and according to the connectance in panel (c). (PDF 17 kb) [file 40168_2018_496_MOESM11_ESM.pdf]

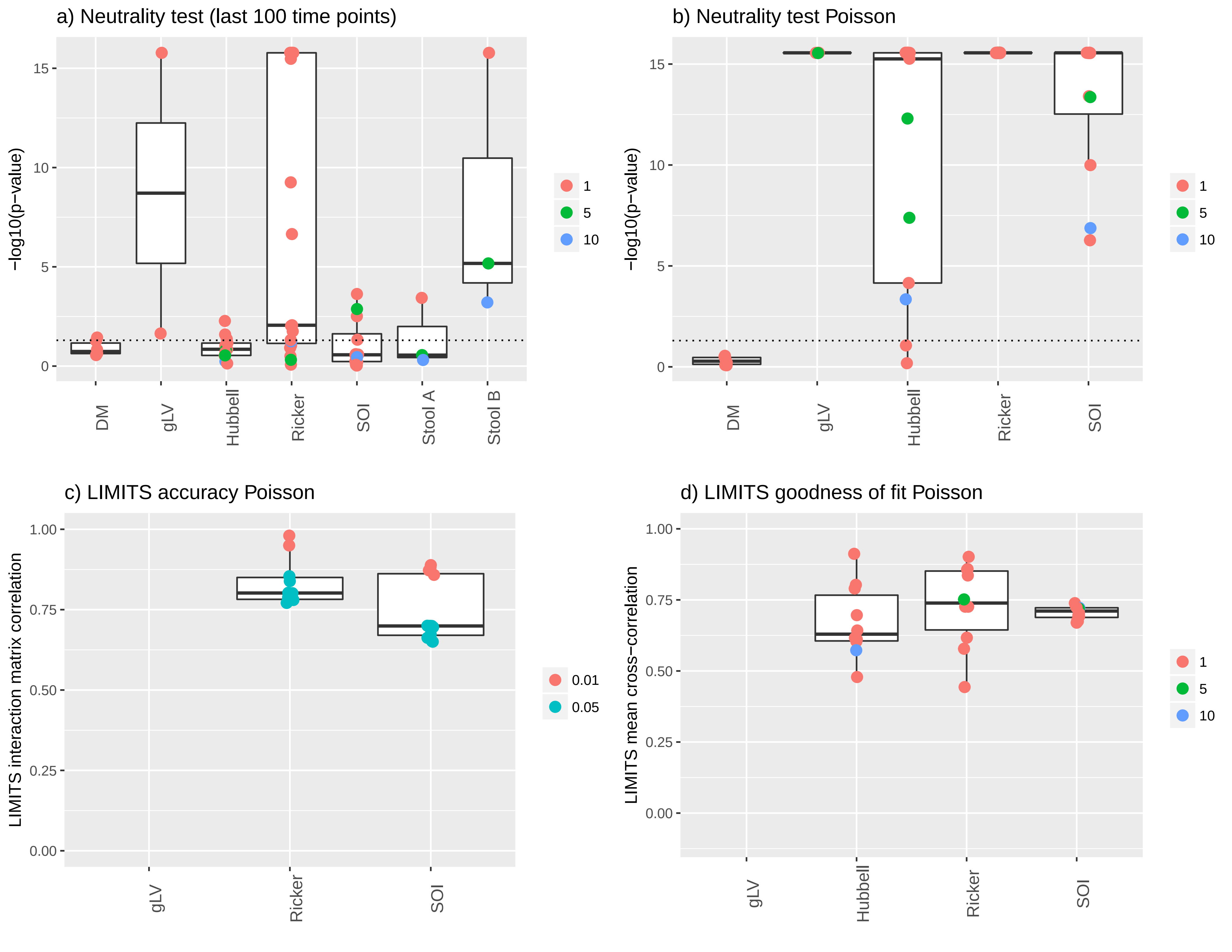

a) Neutrality test (last 100 time points)
−log10(p−value)
0
5
10
15
DM
gLV
Hubbell
Ricker
SOI
Stool A
Stool B
1
5
10
b) Neutrality test Poisson
−log10(p−value)
0
5
10
15
DM
gLV
Hubbell
Ricker
SOI
1
5
10
c) LIMITS accuracy Poisson
LIMITS interaction matrix correlation
0.00
0.25
0.50
0.75
1.00
gLV
Ricker
SOI
0.01
0.05
d) LIMITS goodness of fit Poisson
LIMITS mean cross−correlation
0.00
0.25
0.50
0.75
1.00
gLV
Hubbell
Ricker
SOI
1
5
10
