# Supplementary material for: Signatures of ecological processes in microbial community time series
Source: Microbiome. 2018 Jun 28;6:120. doi: 10.1186/s40168-018-0496-2 (PMC6022718; doi:10.1186/s40168-018-0496-2)
Supplement: Supplementary file 12 — Figure S10. The accuracy of network inference with LIMITS decreases more strongly when applied to the last 100 than to the first 100 time points. (a) LIMITS accuracy, i.e., mean correlation of inferred and known interaction matrix, for the first 100 time points. (b) LIMITS goodness of fit for the first 100 time points. The goodness of fit was computed as the mean correlation between original and predicted time series. (c) LIMITS accuracy for the last 100 time points. Since gLV time series are constant, no network could be inferred for them. (d) LIMITS goodness of fit for the last 100 time points. The correlation between the goodness of fit to the Ricker model and the intrinsic noise strength observed in noise-free time series is lost. The data points are colored according to the connectance in panels (a) and (c), according to interval in panel (b) and according to the intrinsic noise strength sigma in panel (d). (PDF 17 kb) [file 40168_2018_496_MOESM12_ESM.pdf]

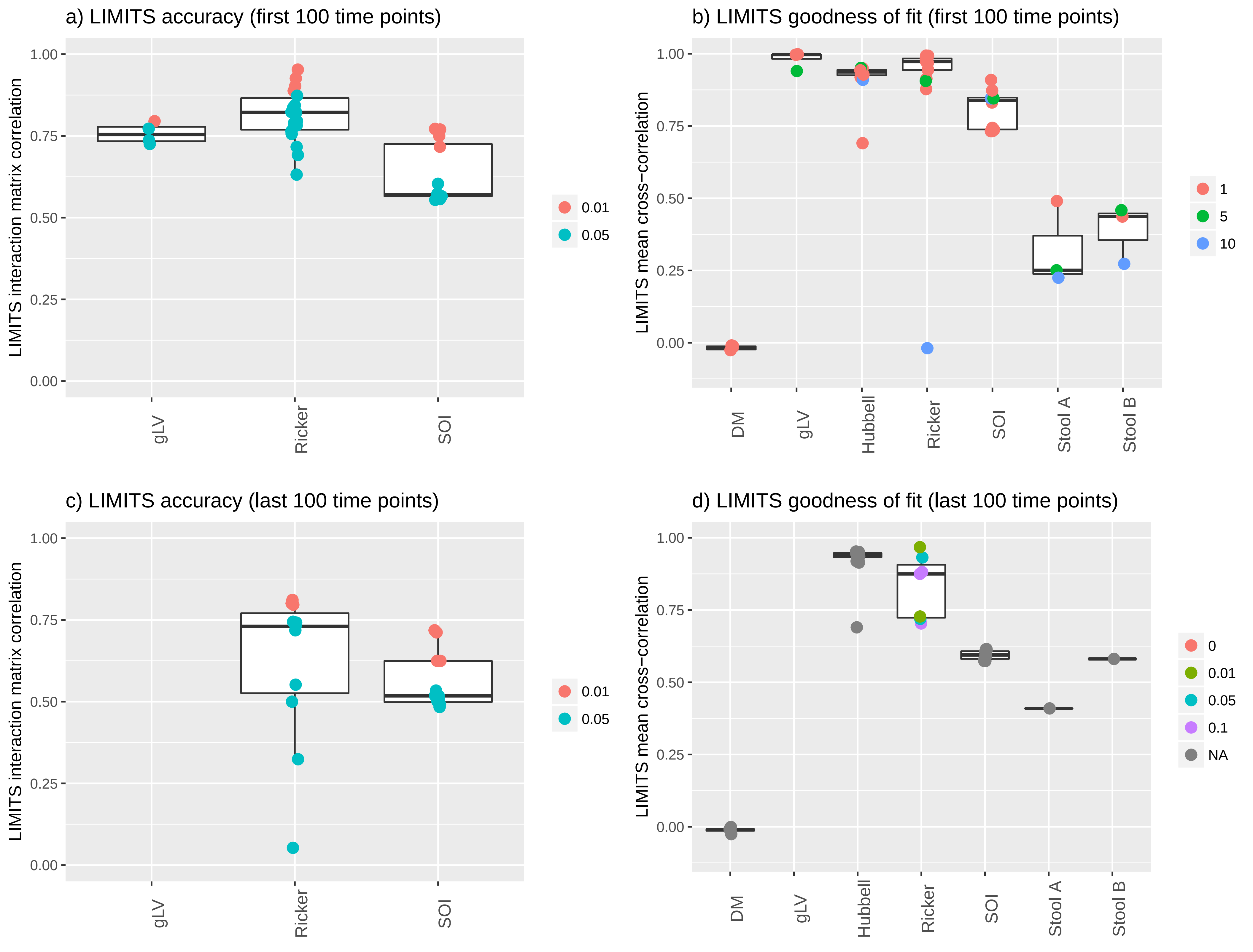

a) LIMITS accuracy (first 100 time points)
LIMITS interaction matrix correlation
1.00
0.75
0.50
0.25
0.00
gLV
Ricker
SOI
0.01
0.05
b) LIMITS goodness of fit (first 100 time points)
LIMITS mean cross−correlation
1.00
0.75
0.50
0.25
0.00
DM
gLV
Hubbell
Ricker
SOI
Stool A
Stool B
1
5
10
c) LIMITS accuracy (last 100 time points)
LIMITS interaction matrix correlation
1.00
0.75
0.50
0.25
0.00
gLV
Ricker
SOI
0.01
0.05
d) LIMITS goodness of fit (last 100 time points)
LIMITS mean cross−correlation
1.00
0.75
0.50
0.25
0.00
DM
gLV
Hubbell
Ricker
SOI
Stool A
Stool B
0
0.01
0.05
0.1
NA
